# Impact of prosthesis oversizing on clinical outcomes of transcatheter aortic valve implantation using a self-expandable Evolut R valve

**DOI:** 10.1186/s43044-024-00450-0

**Published:** 2024-02-12

**Authors:** Mohammed Moustafa Elnwagy, Mahmoud Mohamed Ali Baraka, Mohamed Saber Hafez, Diaa Kamal, Maiy Hamdy El-Sayed, Ahmad E. Mostafa

**Affiliations:** Cardiology department, Ain Shams University, Abbassya, P0 11591, Cairo, Egypt

**Keywords:** Anscatheter aortic valve implantation, Para-valvular leakage, Oversizing index

## Abstract

**Background:**

Transcatheter Aortic Valve Implantation (TAVI) has a growing target population after being indicated even in low-surgical-risk patients with severe symptomatic aortic stenosis. However, postoperative outcomes can be compromised due to para-valvular leakage (PVL). A lot of procedural steps have been investigated to decrease this partially avoidable operational hazard. Oversizing is a main technique to decrease the PVL, despite being itself a risky step. Many studies have been conducted to identify the optimum degree of oversizing. However, studies about oversizing by more than 20% are scarce. We aimed to evaluate the safety and efficacy of oversizing equal to or more than 20%.

**Results:**

209 patients who underwent TAVI using the self-expandable valve Evolut R were initially included. 66 patients were excluded because of the baseline conduction disturbance and lack of sufficient data, so 143 patients, 60 females and 83 males, were enrolled in our study as two groups based on the degree of oversizing: Group A included 97 patients with an oversizing index (OI) of less than 20%, and Group B included 46 patients with an OI of 20% or more. We conducted a new technique for more accurate measuring of the OI in the context of the implantation depth, and our patients were categorized using this technique. Our findings have met our primary end point in terms of the safety and efficacy of oversizing by 20% or more. There was no significant difference between both groups in terms of new-onset conduction disturbance (NOCD), with zero cases of annular rupture or coronary encroachment. In terms of efficacy, The incidence of significant PVL (grade 2 or more) in group B was less than in group A (*P* value 0.007). The ROC curve found that the minimum depth of implantation-derived oversizing (DIDO) to predict no significant PVL was less than 17%.

**Conclusion:**

Prosthesis oversizing by 20% using the self-expandable Evolut R valve is safe and effective, with no significant effect on the conduction system, coronary encroachment, or annular injury, and warrants a greater reduction in the incidence of significant PVL.

## Background

Transcatheter Aortic Valve Implantation (TAVI) has proven itself as an effective treatment for elderly patients with severe symptomatic aortic stenosis with or without high risk profile. However, it’s the preferred strategy in high risk profile patient [1, 2] and it's non inferior to surgery in patients with low risk as reported by recent studies [3, 4].

Despite many advantages over surgical aortic valve replacement, a growing clinical experience with TAVI has revealed several intra- and post-procedure complications that can occur.

Para-valvular leakage (PVL) is one of the most important complications that can occur. Moderate or severe para-valvular leakage can adversely impact the clinical outcome. However, mild PVL can also impact clinical outcomes in the long term [5]. PVL can be avoided recently by many techniques, including optimizing the oversizing degree of the device [6].

Another important complication is the occurrence of postoperative conduction abnormalities due to the mechanical compression of the HIS bundle lying near the aortic annulus [7]. Coronary ostium encroachment is also another important complication that occurs in high-risk patients whose ostium lies less than 10 mm from the aortic annulus. This usually occurs in small sinotublar junction diameters, shallow sinuses, and small aortic annuli due to ostium obstruction by the calcification of the aortic valve or the native leaflets themselves [8].

The incidence of postoperative conduction abnormality and coronary ostium obstruction is both related to patients' factors and device factors. Increasing the size of the device more than it should leads to these complications or even annular rupture, especially with balloon-expanded valves oversizing by more than 20% [8, 9].

The cut off point of over sizing degree with best efficiency (less PVL) and least complication is not yet well established. Some recent studies suggested 14% as safe cut off point, while other has gone up to 17.6% for the self-expandable Evolut system and 10.2% for the balloon expandable system, but there are scarce data about the impact of the oversizing index equal or more than 20% [10, 11].

The Evolut R valve is a retrievable valve giving the advantage of avoiding too deep or too high implantation. It can be aligned with the commissures allowing access for the coronary perfusion and engagement. It also has a supra-annular leaflet design, ensuring wider effective orifice areas (EOAs). It has lengthened the inflow skirt to ensure a longer landing zone and less PVL [12]. A newer generation of self-expandable valve, Evolut R Pro, has been added to this group with the advantage of an outer skirt to prevent even milder para-valvular leakage [13].

The aim of this study was to evaluate the safety and efficacy of oversizing by 20% or more in the context of the depth of implantation using a self-expandable (Evolut R) valve in a low-risk population. Detecting any new-onset conduction disturbance, coronary encroachment, annular injury, and immediate postoperative assessment of PVL were our primary end points. Our secondary end point was to find predictors for new-onset conduction disturbance (NOCD) and para-valvular leakage.

## Methods

### Study population

A total of two hundred and nine (209) consecutive patients with severe symptomatic aortic stenosis who were initially selected based on the recommendations of the heart team at Ain Shams University hospitals for the TAVI procedure as the preferred method of intervention according to the European Society of Cardiology guidelines, using Evolut R valves, were initially included. Approval was obtained from the ethical committee at Ain Shams University before starting the research.66 patients were excluded from the study; 42 patients had underlying conduction disturbances (10 cases with LBBB, 15 cases with RBBB, 7 cases with IVCD, 10 cases with prolonged PR interval), and 21 patients had an evolut valve size 26 implanted. Three patients required evolut valve sizes 23. During the study, we excluded patients who were implanted with Evolut valve sizes 23, and 26, as we could not perform the in vitro measurements due to the unavailable demonstration models (Demos).

The study was proposed as a prospective and retrospective observational cohort study, depending on the registry of the Ain Shams University Hospitals TAVI Heart Team. We initially included 49 patients retrospectively from January 2017 to December 2020 who met our inclusion and exclusion criteria. The rest of the patients were included from January 2021 to March 2023 as prospective. There was a considerable rise in the number of TAVI patients in 2021,2022,and 2023 after the integration of the procedure into the national health insurance system in Egypt.

#### Inclusion criteria

Patients with severe symptomatic aortic stenosis who underwent TAVI using (Evolut R) valve sizes 29 and 34 according to ESC guidelines [14].

#### Exclusion criteria

1. Severe sub-annular calcification (determined by CT imaging in a semi-quantitative method as a single focus extending more than 10 mm)
2. Hypersensitivity or contraindication to any study medication
3. Bicuspid aortic valve
4. Any degree of conduction abnormality (prolonged PR interval, bundle branch block, inter-ventricular conduction delay (IVCD))
5. Previous pacemaker insertion
6. Valve in valve implantation

All patients were counselled about the procedure, and informed consent was obtained. Approval of the Ain Shams University ethical committee was obtained as it conforms to the ethical guidelines of the 1975 Declaration of Helsinki as revised in 2008 [15].

**Fig. 1:**
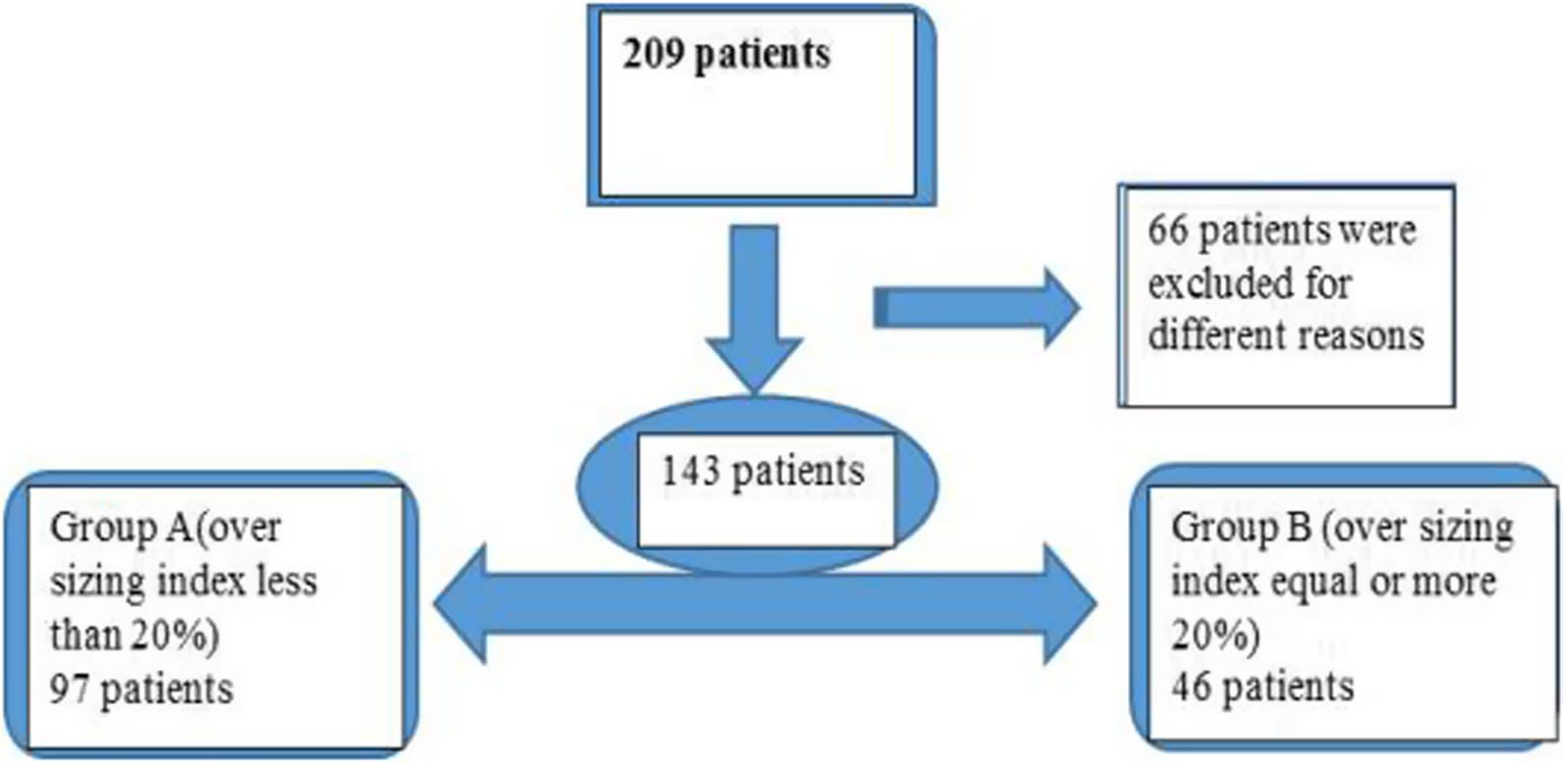
The study population design

We categorized the patient population into two groups based on the degree of oversizing. The first group (A) included those with an oversizing degree less than 20%, and the second group (B) included those with an oversizing degree equal to or greater than 20%, as illustrated in Fig. 1

### Study parameters

#### Pre-interventional parameters

**Fig. 2:**
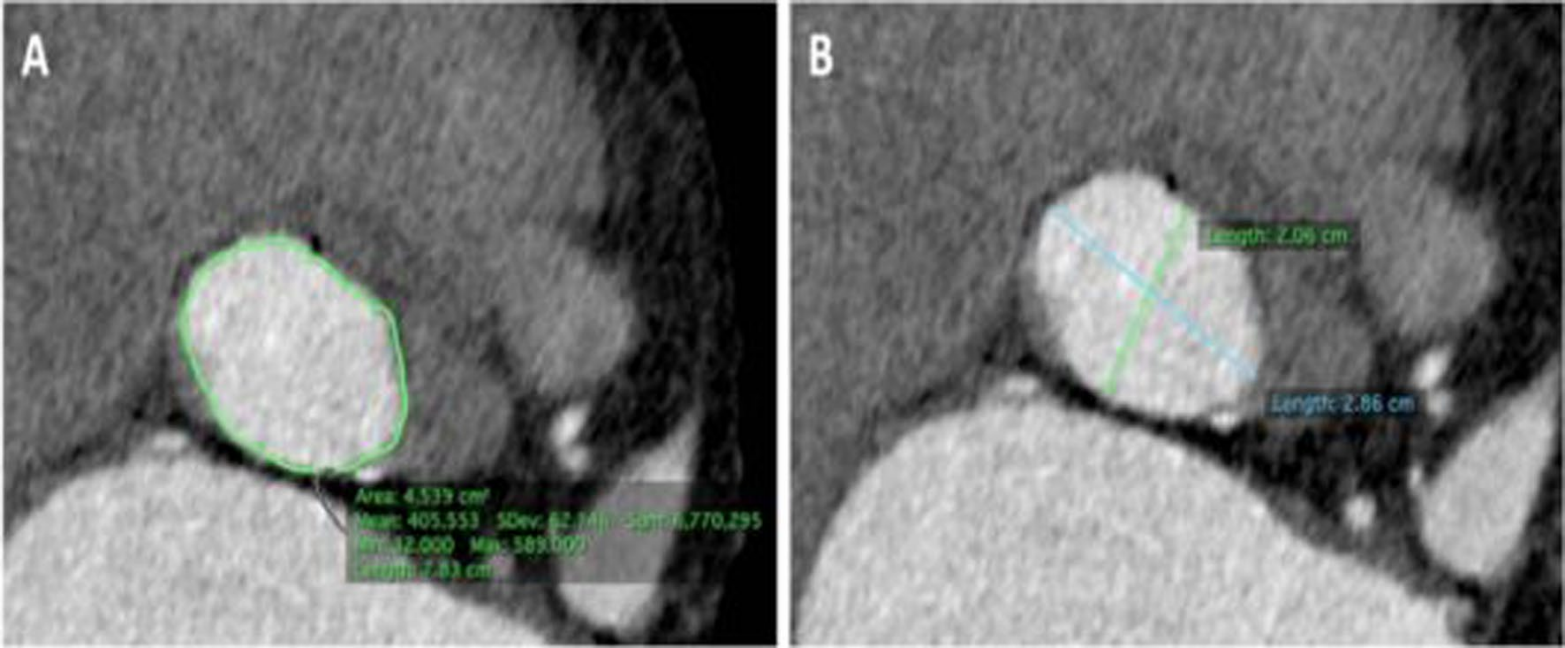
CT-derived measurements of the aortic valve annulus

**Fig. 3:**
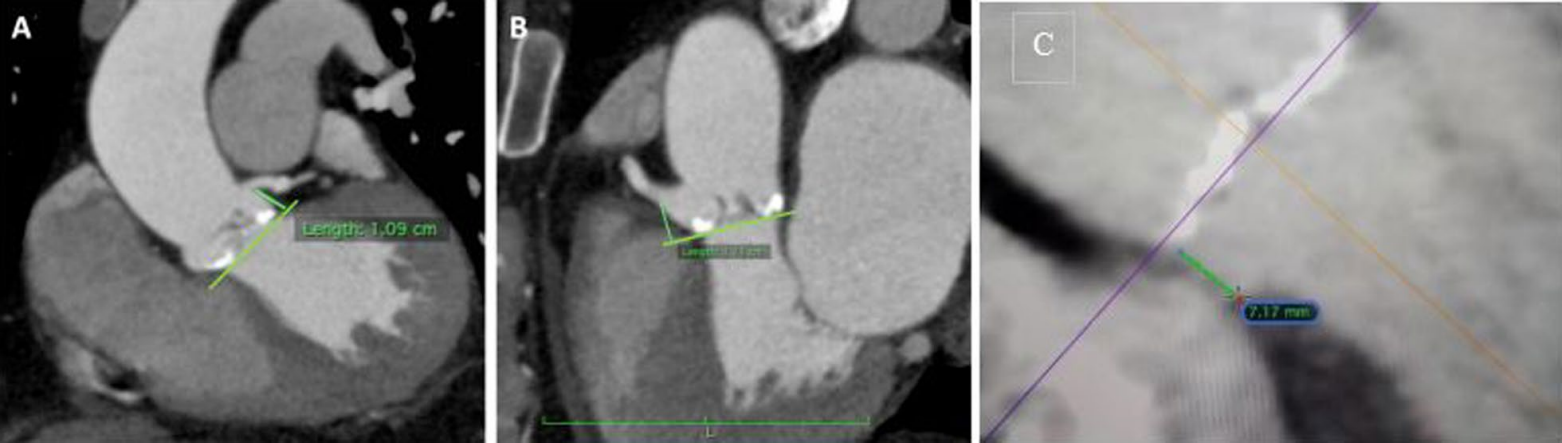
CT-derived measurements of heights of the coronary arteries Ostia from the aortic annulus and membranous septum length

**Fig. 4:**
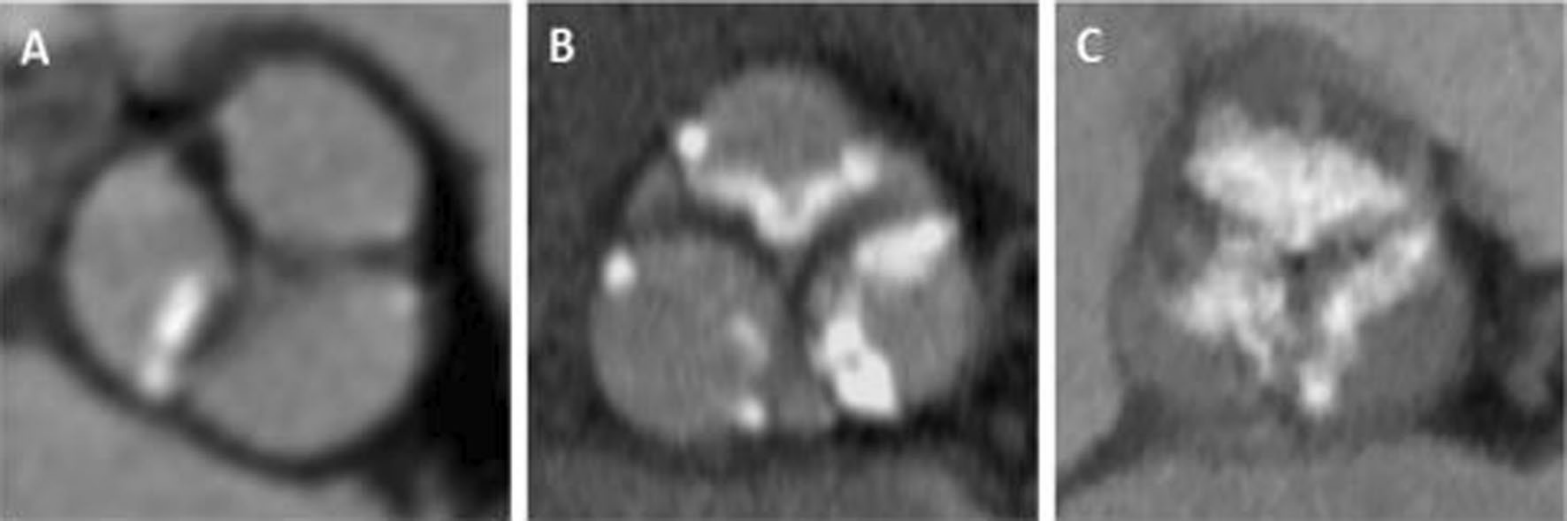
Example of the grading of Aortic valve calcification distribution among the study population according to device landing zone classification

A- **Medical history parameters** included** a**ge,sex,body mass index, baseline operative risk of the patients estimated by the logistic Euro score II and STS score [16], and past medical history.
B- **Electrocardiogram (ECG) parameters included**: basic rhythm(paroxysmal AF was recognized by reviewing previous ECG or past medical history),PR interval,QRS duration,and bundle branch block**.**
C- **Transthoracic echocardiography parameters** By General Electric Vivid S5 Healthcare (9900 Innovation Drive, Wauwatosa, WI 53226, **U.S.A.)** devices using the M5S probe and Philips CX50 bedside machines (Philips Medical Systems, Bothell, WA, USA) using the S5-1 probe, the following standard measurements were estimated according to American Society of Echocardiology (ASE) guidelines[17]:diameters of the aortic annulus at the mid systole, gradient across the aortic valve: mean and peak pressure gradient using AV tracing of the aortic jet obtained by CW signal, valve morphology: Bicuspid versus tri-leaflet valve, degree of AR classified as absent (grade 0), mild (grade 1), moderate (grade 2–3), and severe (grade 4) AR, aortic valve area (using continuity equation), LV internal dimensions (end-diastolic and end-systolic), LV wall thickness (septum and posterior wall), LV ejection fraction (using M mode and Simpson's method), and estimated right ventricular pressures.
D- **Computerized tomography (CT) pre-TAVI protocol parameters All** CT images were obtained by a 256-detector dual tube-multi slice (MS) CT scanner (SOMATOM Definition Flash; Siemens Healthineers, Erlangen, Germany) and processed by two observers using Osirix 9.5 MD version software to acquire 3D reconstruction to obtain the following: Aortic annulus found at the level of the nadirs of cusp attachments (measuring the diameters (minimum, maximum and mean diameter), area, and perimeter) as shown in Fig. 2, heights of the coronary arteries Ostia from the aortic annulus, membranous septum length as shown in Fig. 3,diameters of sinuses of Valsalva,basal septal calcification as present or absent, angle of the aortic root to identify the horizontal aorta as more than 50 degree from horizontal axis in the coronal section, projected angles of fluoroscopy co-planar view and cusp overlap view,and the degree of aortic valve calcification graded according to the device landing zone (DLZ) classification, as follows: Grade 1: single or two spots of increased brightness. Grade 2: scattered spots of increased brightness were confined to leaflet margins. Grade 3: heavy calcification with some time commissures crossing by the calcium; Grade 4: massive calcification outreaching to the aortic annulus ± left ventricular outflow tract (LVOT) [18], as shown in Fig. 4

### Procedural details, and procedural variables

The procedure was performed with local anesthesia in combination with a mild systemic sedative and analgesic treatment. Vascular access was obtained percutaneously through the common femoral artery (a surgical cut down was performed in 109 patients, and the Perclose ProGlideTM Suture-Mediated Closure System successfully closed the vascular access in 34 patients, depending on the availability and feasibility of the device use).

Invasive cardiac evaluation with coronary angiography was done if the coronary anatomy by CT pre-TAVI protocol suggested a high probability of coronary artery disease (e.g., high degree of coronary calcification) or other medical condition (e.g., recent acute coronary syndrome).Lower abdominal aortography, if needed, was also done before the start of the procedure to determine the common femoral artery puncture site before preclose proGlide use, especially if good Doppler signals couldn't be obtained due to excessive body weight.

A transient pacemaker lead was introduced through a jugular sheath and placed in the RV to allow pacing during valve deployment (up to 100 bpm) or rapid pacing during balloon valvuloplasty (180–220 bpm and a drop in the systolic blood pressure less than 50 mmHg and a pulse pressure less than 10 mmHg) [19]. Balloon valvuloplasty under rapid pacing was performed before device placement in 36 patients due to severe aortic calcification and the high probability of under-deployment of the device, after which, over a stiff guide wire placed in the left ventricle, the device was deployed under fluoroscopic guidance. Hemodynamic outcomes were assessed continuously during the procedure.

For bioprosthesis size selection, previously published literature and the manufacturer-recommended CT-based sizing algorithm (23 mm prosthesis for annuli 18–20 mm; 26 mm prosthesis for annuli 20–23 mm; 29 mm prosthesis for annuli 23–26 mm; 31 mm/34 mm prosthesis for annuli 26–29 mm/26–30 mm) were taken into consideration and implanted at a depth of less than 6 mm, performing a degree of oversizing in almost every patient according to the current practice [10, 11, 20, 21].

Post-implantation balloon valvuloplasty under rapid pacing was performed in 25 patients due to a significant mean pressure gradient across the implanted valve of more than 10 mmHg or in cases of significant para-valvular leakage (PVL) assessed by trans thoracic echocardiography.

### Post-intervention parameters

Evaluation of immediate post-procedural variables was performed using a aortic root angiogram and echocardiography (TTE).

**Fig. 5:**
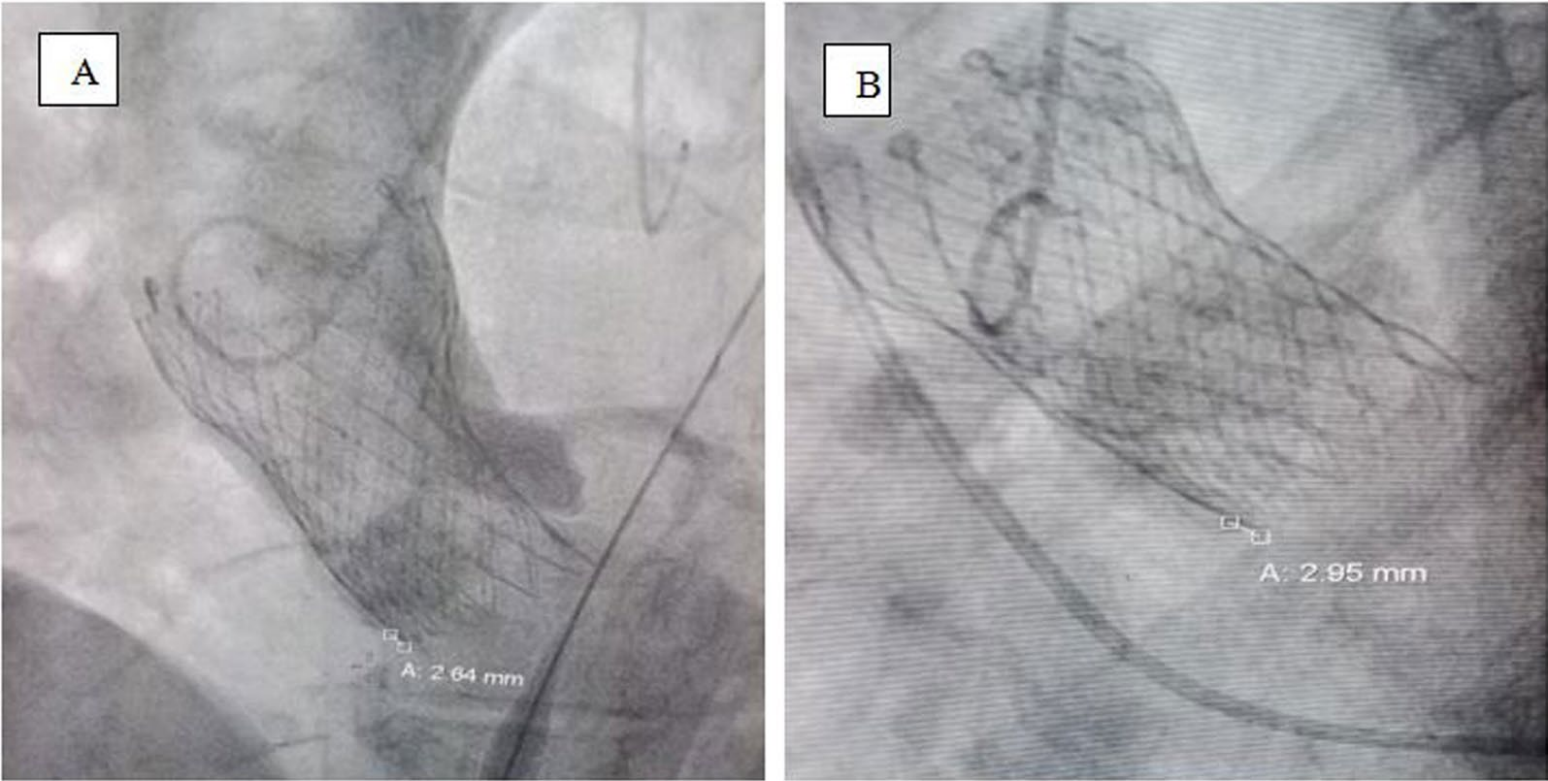
Example of Cine Aortography for measurement of the post implantation depth

**Fig. 6:**
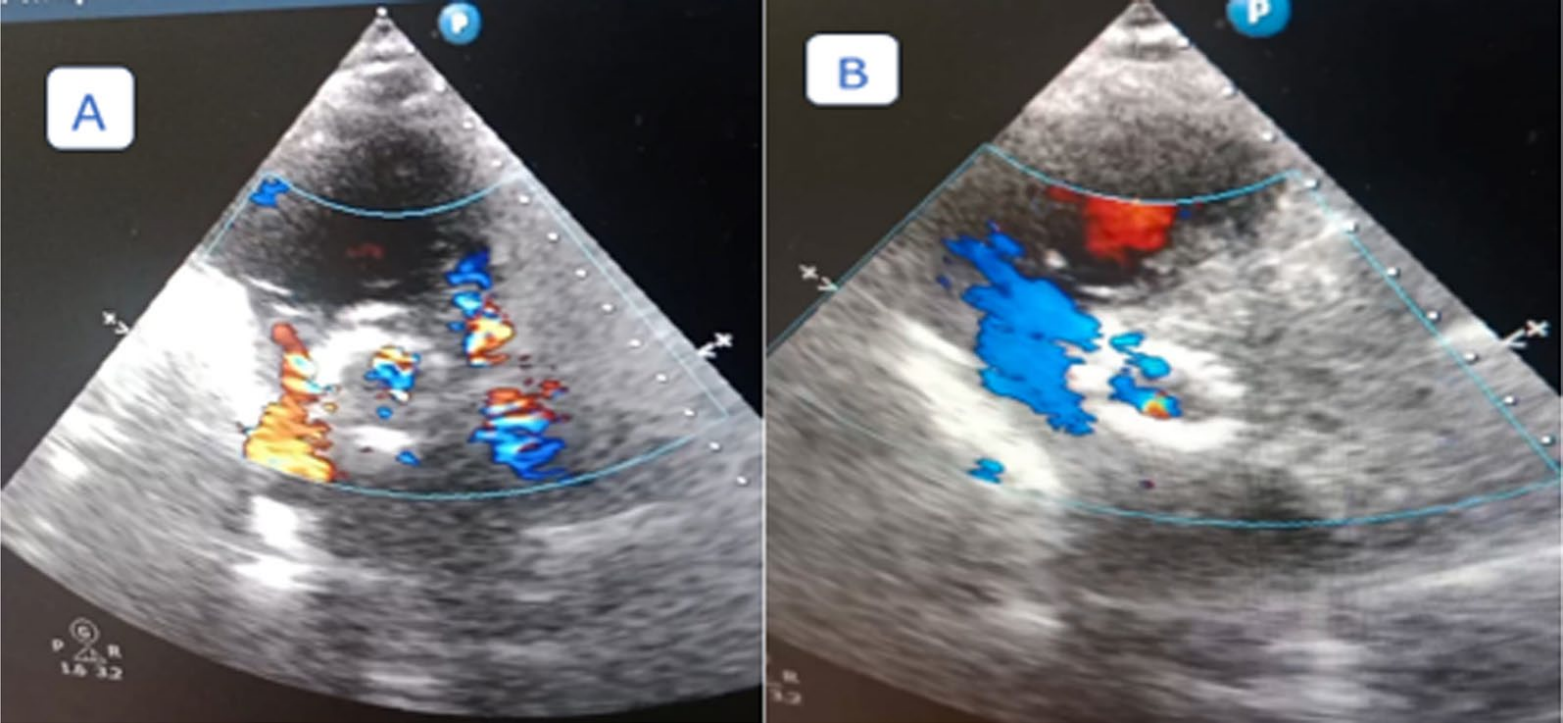
Example of PVL assessment post implantation by bedside TTE

A- Valve depth of implantation (DI): this was measured after implantation by aortography in order to avoid the valve parallax, usually in cusp overlap view with minor (left anterior oblique) LAO modification. We measured the implantation depth from the lower limit of the NCC to the lowermost part of the valve frame. (Fig. 5**)**
B- Depth of implantation/membranous septum (DIMS): We calculated this parameter by measuring the depth of implantation by fluoroscopy and dividing it by the membranous septum from CT parameters [22].
C- The para-valvular Leakage (PVL): was graded by a four-class grading system using bedside TTE according to the circumferential extent of the PVL by color Doppler in a short-axis view as follows: Grade 1 (trace or trivial): represents less than 10% of the circumference of the annular ring. Grade 2 (mild): represented 10–20% of the circumference of the annular ring. Grade 3 (moderate): represented 20–30% of the circumference of the annular ring. Grade 4 (severe): represented as equal to or more than 30% of the circumference of the annular ring [23]. The differentiation between grade 3 and grade 4 was based on other parameters (vena contracta (VC) up to 6 mm was graded as grade 3, while VC greater than 6 mm was graded as grade 4). The para-valvular leakage grading measured in our study was done after any trial of post-implantation dilatation as a final result. Grade 2 or more was considered significant PVL ([5]. The assessment of PVL was done immediately postoperatively and another time during the hospital stay before discharge, usually within 2–3 days postoperatively (Fig. 6**).**

### The degree of perimeter oversizing using Evolut R valve in the context of the implantation depth

To the best of our knowledge, there is no available data about the outer diameter of the valve at different heights from its base but only the diameter at its outer rims and middle waists. Traditionally, to calculate the degree of oversizing, the nominal perimeter at the inflow rim is used in the following equation: Over-sizing = [(device perimeter at its inflow rim minus annulus perimeter) / annulus perimeter] × 100. Measuring the exact outer diameter of the valve that comes into contact with the aortic annulus after deployment, depending on the length of the depth of implantation, helps us get a more accurate measurement of the oversizing index. So we had to measure the outer diameter of the Evolut R valve through its 8-mm length from its inflow base, as there is no uniform diameter through its length due to the conical nature of the prosthesis. We could only collect data about the different diameters at different heights for valve sizes 29 and 34 because the demos we could get from the manufacturer were of those sizes Table 1.

**Table 1:** Shows the diameters of (Evolut R) valves 29 and 34 at different depth of implantation from 1 to 8 mm

Depth of implantation (mm)	Corresponding diameter of outer rim valve size 29	Depth of implantation (mm)	Corresponding diameter of outer rim valve size 34
1	28.65	1	33.75
2	28.35	2	33.40
3	28.00	3	32.20
4	27.80	4	31.80
5	27.50	5	31.10
6	27.30	6	30.90
7	27.00	7	30.40
8	26.80	8	30.00

**Fig. 7:**
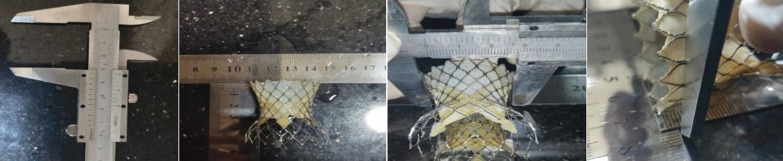
The manual method used for measuring the annulus of the Evolut R valves at different heights from its base using Vernier calliper

We used a simple and accurate manual method that was repeated several times by different people using two rulers perpendicular to each other and a Vernier caliper (Figs. 6 and 7). After measuring the outer diameters at different heights from each valve base, we calculated the nominal perimeter at different depths of implantation for each valve size. Finally, a more accurate method than before was used to calculate the degree of oversizing, as follows: Over-sizing = [(device perimeter at the depth of implantation minus annulus perimeter) / annulus perimeter] × 100. Based on our modified method of calculating the oversizing index, we categorized our population into two main groups: group A included those with DIDO% less than 20%, and group B included those with DIDO% equal to or more than 20%.

**Fig. 8:**
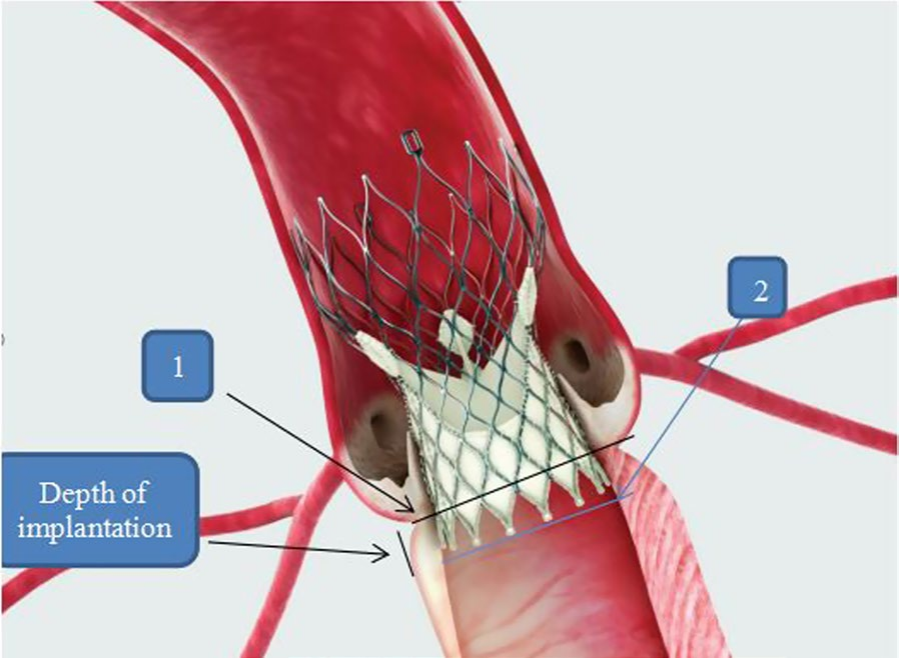
The difference between the traditional method and the DIDO method for calculating oversizing index (OI)

Figure 8 The difference between the traditional method and the DIDO method for calculating OI using different nominal device perimeters with nominal perimeter at line 1 used for the DIDO method and nominal perimeter at line 2 used for the traditional method

### Statistical analysis

Continuous variables with normal distribution are presented as mean ± standard deviation and range, whilst those with non-normal distribution are reported as median and interquartile range (IQR). Continuous variables were compared by Student’s t-test or *Mann–Whitney test* as appropriate, while categorical variables were compared by chi-square tests. Statistical analysis was performed using SPSS 25 for Windows (SPSS Inc, Chicago, Illinois, USA). *The receiver operating characteristic curve (ROC)* was used in quantitative form to determine sensitivity, specificity, positive predictive value (PPV), negative predictive value (NPV), and area under curve (AUC) of DIDO to predict PVL of the studied patients and of factors statistically significant associated with NOCD to find cut-off points. Binary* logistic regression analysis* in the form of uni-variate and multivariate was done to assess the predictors of conduction disturbance and PVL with their odds ratio (OR) and 95% confidence interval (CI). The confidence interval was set to 95%, and the margin of error accepted was set to 5%. So, the p-value was considered significant if it was < 0.05.

## Results

### Baseline characteristics (Table 2)

**Table 2:** Showing the descriptive data, clinical, ECG, echocardiographic and CT parameters

Demographics		No. = 143
Age (years)	Female	60 (42.0%)
	Male	83 (58.0%)
Weight (Kg)	Mean ± SD	73.80 ± 6.55
	Range	55–88
Height (m)	Mean ± SD	79.30 ± 14.36
	Range	50–125
BMI (Kg/m^2^)	Mean ± SD	1.65 ± 0.08
	Range	1.5–1.82
BSA(m^2^)	Mean ± SD	29.07 ± 5.18
	Range	18.82–47.87
Age (years)	Mean ± SD	1.87 ± 0.18
	Range	1.44–2.39

Clinical data		No. = 143
CABG	No	130 (90.9%)
	Yes	13 (9.1%)
Euro score II %	Median (IQR)	3.45 (1.99–5.43)
	Range	0.84–40.2
STS PROM %	Median (IQR)	2.45 (1.73–3.54)
	Range	0.66–16.98
Smoking	Non-smoker	107 (74.8%)
	Smoker	25 (17.5%)
	EX -smoker	11 (7.7%)
DM	No	75 (52.4%)
	Yes	68 (47.6%)
HTN	No	33 (23.1%)
	Yes	110 (76.9%)
RHD	No	136(95.1%)
	Yes	7(4.9%)
IHD	No	87 (60.8%)
	Yes	56 (39.2%)
CVS	No	134 (93.7%)
	Yes	9 (6.3%)
CrCl (ml/min)	Mean ± SD	63.46 ± 26.73
	Range	9–161
CLD	No	132 (92.3%)
	Yes	11 (7.7%)

Electrocardiographic data		No. = 143	
AF	No	117 (81.8%)	
	Permanent	16 (11.2%)	
	Paroxysmal	10 (7.0%)	
PR interval (mm)	Mean ± SD	162.19 ± 36.11	
	Range	0–200	
QRS complex (mm)	Mean ± SD	88.67 ± 9.44	
	Range	80–100	
Echo-data		No. = 143	
EF(%)	Mean ± SD	60.25 ± 13.14	
	Range	20–84	
SWT (mm)	Mean ± SD	12.64 ± 1.78	
	Range	8–18	
SWTi (mm/m^2^)	Mean ± SD	6.74 ± 1.17	
	Range	0.51–9.8	
PWT (mm)	Mean ± SD	12.25 ± 1.63	
	Range	7–16	
PWTi(mm/m^2^)	Mean ± SD	6.53 ± 1.13	
	Range	0.51–9.8	
LVEDD (mm)	Mean ± SD	50.35 ± 6.57	
	Range	35–74	
LVEDDi (mm/m^2^)	Mean ± SD	26.89 ± 4.76	
	Range	0.51–46.54	
LVESD (mm)	Mean ± SD	33.39 ± 7.49	
	Range	20–63	
LVESDi (mm/m^2^)	Mean ± SD	17.89 ± 4.73	
	Range	0.51–37.73	
PPG (mmHg)	Mean ± SD	81.82 ± 21.79	
	Range	35–171	
MPG (mmHg)	Mean ± SD	50.01 ± 13.80	
	Range	20–100	
AVA (cm^2^)	Median (IQR)	0.7 (0.5–0.8)	
	Range	0.2–1	
AVAi (cm^2^/m^2^)	Mean ± SD	0.38 ± 0.11	
	Range	0.1–0.59	
AR	0	15 (10.5%)	
	1	86 (60.1%)	
	2	38 (26.6%)	
	3	4 (2.8%)	
	4	(0.0%)	
RVSP (mmHg)	Mean ± SD	42.01 ± 11.01	
	Range	20–86	
CT data		No. = 143	
Annulus. Diameter (mm)	Mean ± SD	24.17 ± 1.73	
	Range	20–28.4	
Annulus. Diameter.indexed (mm/m^2^)	Mean ± SD	12.99 ± 1.40	
	Range	8.93–17.48	
Annulus. Perimeter (mm)	Mean ± SD	77.97 ± 5.09	
	Range	69–92	
Annulus. Perimeter. Indexed (mm/m^2^)	Mean ± SD	41.89 ± 4.33	
	Range	34.17–56.6	
Annulus. Area (mm^2^)	Mean ± SD	460.84 ± 62.79	
	Range	339–666	
Annulus. Area indexed (mm^2^/m^2^)	Mean ± SD	247.42 ± 37.40	
	Range	166.91–377	
LMCA.(mm)	Mean ± SD	12.96 ± 2.61	
	Range	6.6–20.5	
LMCAi (mm/m^2^)	Mean ± SD	6.96 ± 1.51	
	Range	3.75–11.58	
RCA.(mm)	Mean ± SD	14.95 ± 3.10	
	Range	9.2–26.3	
RCAi (mm/m^2^)	Mean ± SD	8.06 ± 1.94	
	Range	4.62–14.85	
MS.(mm)	Mean ± SD	8.82 ± 2.05	
	Range	5–15.3	
MS I (mm/m^2^)	Mean ± SD	4.76 ± 1.27	
	Range	2.58–8.82	
Grade of aortic Calcification	Grade 1	1	0.7%
	Grade 2	24	16.8%
	Grade 3	59	41.3%
	Grade 4	59	41.3%
Severe or non severe aortic Calcification	Grade 1–3	84	58.7%
	Grade 4	59	41.3%
Septal calcification	No	123	86.0%
	Yes	20	14.0%

*AF* Atrial fibrillation, *AR* Aortic regurgitation, *AVA* Aortic valve area, *AVAi* Aortic valve area indexed, *BMI* Body mass index, *BSA* Body surface area, *Ca* Calcification, *CABG* Coronary artery bypass graft, *CLD* Chronic lung disease, *CrCl* Creatinine clearance, *CVS* Cerebrovascular stroke, *DM* Diabetes mellitus, *EF* Ejection fraction, *HTN* Hypertension, *IHD* Ischemic heart disease, *LVEDD* Left ventricle end diastolic dimension, *LVEDDi* Left ventricle end diastolic dimension indexed, *LVESD* Left ventricle end systolic dimension, *LVESDi* Left ventricle end systolic dimension indexed, *LMCA* Left main coronary artery height, *LMCAi* Left main coronary artery height indexed, *MPG* Mean pressure gradient, *MS* Membranous septum, *MSi* Membranous septum indexed, *PWT* Posterior wall thickness, *PWTi* Posterior wall thickness indexed, *PPG* Peak pressure gradient, *RCA* Right coronary artery height, *RCAi* Right coronary artery height indexed, *RHD* Rheumatic heart disease, *RVSP* Right ventricular systolic pressure, *STS PROM* Society of thoracic surgery predicted risk of mortality, *SWT* Septal wall thickness, *SWTi* Septal wall thickness indexed

The study population included 60 (42.0%) females and 83 (58.0%) males of a total of 143 patients, with a mean age of 73.8 years and a range of 55–88 years old. The average BMI was 07 ± 5.18 kg/m^2^. The average BSA was 1.87 ± 0.18. Concerning the clinical characteristics, 13 patients (9.1%) had CABG before TAVI operations, 25 patients (17.5%) were smokers, and 11 patients (7.7%) were ex-smokers. 68 patients (47.6%) were diabetic, 110 patients (76.9%) were hypertensive, 56 patients (39.2%) had IHD, 9 patients (6.3%) had CVS, 11 patients (7.7%) had chronic lung disease, and only 7 patients (4.8%) had RHD. The average CrCl was 63.46 ± 26.73 ml/min.

### Pre-operative ECG, echocardiography, and CT data (Table 2)

The electrocardiographic data showed that 16 patients (11.2%) had permanent AF, while 10 patients (7.0%) had paroxysmal AF. The average PR interval was 162.19 ± 36.11 ms with a range of 0–200 ms, and the average QRS complex was 67 ± 9.44 ms with a range of 80–100 ms.

The echocardiographic measurement revealed that the average EF was 60. 25 ± 13.14% (20–84%), the average indexed aortic valve area ( AVAi) was 0.38 ± 0.11 cm^2^ with a range of 0.1–0.69 cm^2^, the average PPG was 81.82 ± 21.79 mmHg,the average MPG was 50.01 ± 13.80 mmHg, and the mean RVSP was 42.01 ± 11.01 mmHg. 15 patients (10.5%) did not have any degree of AR, 86 patients (60.1%) had grade I/IV AR, 38 patients (26.6%) had grade II/IV AR, 4 patients (2.8%) had grade III/IVAR.

The CT pre-TAVI protocol illustrated the vascular access and the anatomical characteristics of the aortic annulus. The average aortic perimeters were 77.97 ± 5.09 mm, with an indexed area range of 166.91–377 mm^2^/m^2^. The average LM coronary height was 12.96 ± 2.61 mm, and the average RCA height was 14.95 ± 3.10 mm.The membranous septum length ranged from 5 to 15.3 mm.1 patient (0.7%) was graded as grade 1 and 24 patients 16.8% was graded as grade 2. Both grade 3 and grade 4 aortic valve calcification represented 41.3% each.Only 20 patients (14%) had basal septal calcification.

### Operative and postoperative data (Table 3)

**Table 3:** Showing the descriptive data of operative and postoperative parameters

Operative data		No. = 143
DI (mm)	Median (IQR)	3.1 (2.3–4.85)
	Range	1–8
DI I (mm/m^2^)	Median (IQR)	1.69 (1.25–2.43)
	Range	0.42–4.6
Pre- implantation Dilatation	No	107 (74.8%)
	Yes	36 (25.2%)
Post implantation-Dilatation	No	118 (82.5%)
	Yes	25 (17.5%)
DIMS %	Median (IQR)	36.14 (24.62–59.62)
	Range	10–114.23

ECG post operative		No.	%
Conduction disturbance	No	113	79.0%
	Yes	30	21.0%
Permanent CHB	No	141	98.6%
	Yes	2	1.4%
Transient conduction. Disturbance	No	132	92.3%
	Yes	11	7.7%
New LBBB	No	119	83.2%
	Yes	24	16.8%
QRS widening	No	114	79.7%
	Yes	29	20.3%
PR Prolongation	No	137	95.8%
	Yes	6	4.2%
New onset AF	No	140	97.9%
	Yes	3	2.1%

Postoperative data		No. = 143
DIDO %	Mean ± SD	16.55 ± 5.93
	Range	0–30
PVL	No (0)	61 (42.7%)
	Trivial (1)	68 (47.6%)
	Mild (2)	12 (8.4%)
	Moderate ( 3)	2 (1.4%)
	Severe (4)	0 (0.0%)
Coronary obstruction or annular rupture	No	143 (100.0%)

*AF* Atrial fibrillation, *CHB* Complete heart block, *DI* Depth of implantation, *DIi* Depth of implantation indexed, *DIMS* Depth of implantation to membranous septum ratio, *LBBB* Left bundle branch block, *DIDO* Depth of implantation derived oversizing index, *PVR* Para-valvular leakage

The operative and postoperative data revealed that the median depth of implantation DI was 3.1 mm with an IQR of 2.3–4.85. 36 patients (25.2%) required balloon pre-dilatation, while 25 patients (17.5%) required post-dilatation. The average depth of implantation-derived oversizing index was 16.55 ± 5.9%, with a range from 0 to 30%. There was no coronary affection in any case in the study population. The post-operative ECG measurements found that 30 patients (20.9%) suffered from some sort of conduction disturbance such as new-onset LBBB, QRS widening, and PR prolongation, with only 2 cases required a permanent pacemaker. 3 patients (2.1%) developed new-onset AF. The cases required a permanent pacemaker belonged to group A with an OI of less than 20%. The final results of the operations in regards to PVL incidence showed that 68 patients (47.6%) showed grade 1 PVL, 12 patients (8.4%) showed grade 2 PVL, and 2 patients (1.4%) showed grade 3 (moderate) PVL. No severe or grade 4 PVL was detected postoperative.

### The descriptive data comparison between group A and group B (Table 4)

**Table 4:** The descriptive data, clinical, ECG, echocardiographic, CT parameters comparison between group A and the extreme oversizing group B

Demographics &clinical data	Group A (< 20%)	Group B (≥ 20%)		Test value	*P*-value	Sig.
	No. = 97	No. = 46				
Age (years)	Female	39 (40.2%)	21 (45.7%)	0.380*	0.538	NS
	Male	58 (59.8%)	25 (54.3%)			
Weight (kg)	Mean ± SD	74.33 ± 7.05	72.70 ± 5.24	1.398•	0.164	NS
	Range					
Height (m)	Mean ± SD	55–88	62–86	0.697•	0.487	NS
	Range	50–115	50–125			
BMI(kg/m^2^)	Mean ± SD	1.65 ± 0.08	1.63 ± 0.07	1.697•	0.092	NS
	Range	1.5–1.82	1.5–1.8			
BSA (m^2^)	Mean ± SD	28.86 ± 5.28	29.53 ± 4.98	− 0.727•	0.468	NS
	Range	18.82–47.87	21.8–41.52			
Age (years)	Mean ± SD	1.88 ± 0.17	1.86 ± 0.19	0.479•	0.633	NS
	Range	1.44–2.25	1.52–2.39			
CABG	No	86 (88.7%)	44 (95.7%)	1.846*	0.174	NS
	Yes	11 (11.3%)	2 (4.3%)			
Euro score II %	Median (IQR)	3.56 (2.28–6.18)	2.97 (1.54–3.89)	− 2.358‡	0.018	S
	Range	0.84–40.2	0.92–8.12			
STS score %	Median (IQR)	2.58 (1.81–3.88)	2.26 (1.57–2.87)	− 2.066‡	0.039	S
	Range	0.66–16.98	0.99–7.08			
Smoking	Non-smoker	71 (73.2%)	36 (78.3%)	0.960*	0.619	NS
	Smoker	19 (19.6%)	6 (13.0%)			
	EX -smoker	7 (7.2%)	4 (8.7%)			
DM	No	51 (52.6%)	24 (52.2%)	0.002*	0.964	NS
	Yes	46 (47.4%)	22 (47.8%)			
HTN	No	23 (23.7%)	10 (21.7%)	0.068*	0.794	NS
	Yes	74 (76.3%)	36 (78.3%)			
RHD	No	94 (96.9%)	42 (91.3%)	2.104	0.147	NS
	Yes	3 (3.1%)	4 (8.7%)			
IHD	No	54 (55.7%)	33 (71.7%)	3.382*	0.066	NS
	Yes	43 (44.3%)	13 (28.3%)			
CVS	No	91 (93.8%)	43 (93.5%)	0.006*	0.938	NS
	Yes	6 (6.2%)	3 (6.5%)			
CrCl (ml/min)	Mean ± SD	61.20 ± 24.39	68.22 ± 30.85	− 1.473•	0.143	NS
	Range	9–134.07	15–161			
CLD	No	89 (91.8%)	43 (93.5%)	0.131*	0.718	NS
	Yes	8 (8.2%)	3 (6.5%)			

ECG	Group A (< 20%)	Group B (≥ 20%)		Test value	P-value	Sig.
	No. = 97	No. = 46				
AF	No	80 (82.5%)	37 (80.4%)	0.303*	0.859	NS
	Permanent	11 (11.3%)	5 (10.9%)			
	Paroxysmal	6 (6.2%)	4 (8.7%)			
PR (ms)	Mean ± SD	163.33 ± 37.09	159.76 ± 34.24	0.521•	0.603	NS
	Range	0–200	80–200			
QRS (ms)	Mean ± SD	89.38 ± 9.55	87.17 ± 9.11	1.310•	0.192	NS
	Range	80–110	80–100			

Echo	Group A (< 20%)	Group B (≥ 20%)		Test value	*P*-value	Sig.
	No. = 97	No. = 46				
EF(%)	Mean ± SD	59.33 ± 13.58	62.17 ± 12.09	− 1.207•	0.229	NS
	Range	20–84	25–78			
SWT (mm)	Mean ± SD	12.73 ± 1.64	12.45 ± 2.04	0.862•	0.390	NS
	Range	8–17	8–18			
SWTi (mm/m^2^)	Mean ± SD	6.76 ± 1.23	6.70 ± 1.04	0.285•	0.776	NS
	Range	0.51–9.8	4.79–8.94			
PWT (mm)	Mean ± SD	12.39 ± 1.49	11.95 ± 1.87	1.519•	0.131	NS
	Range	8–16	7–16			
PWTi(mm/m^2^)	Mean ± SD	6.59 ± 1.20	6.42 ± 0.96	0.796•	0.428	NS
	Range	0.51–9.8	4.29–8.33			
LVEDD(mm)	Mean ± SD	50.58 ± 7.02	49.87 ± 5.56	0.604•	0.547	NS
	Range	35–74	39–64			
LVEDDi (mm/m^2^)	Mean ± SD	26.86 ± 5.23	26.94 ± 3.62	− 0.097•	0.923	NS
	Range	0.51–46.54	19.7–36.99			
LVESD(mm)	Mean ± SD	33.71 ± 7.59	32.72 ± 7.32	0.737•	0.463	NS
	Range	20–63	20–51			
LVESDi(mm/m^2^)	Mean ± SD	17.99 ± 4.88	17.67 ± 4.47	0.379•	0.705	NS
	Range	0.51–37.73	11.08–29.48			
PPG(mmHg)	Mean ± SD	81.99 ± 20.88	81.48 ± 23.80	0.129•	0.897	NS
	Range	38–171	35–145			
MPG (mmHg)	Mean ± SD	50.45 ± 13.59	49.09 ± 14.33	0.549•	0.584	NS
	Range	20–100	25–90			
AVA (cm^2^)	Median (IQR)	0.7 (0.5–0.8)	0.7 (0.5–0.8)	− 0.513‡	0.608	NS
	Range	0.2–1	0.3–8			
AVAi (cm^2^/m^2^)	Mean ± SD	0.37 ± 0.11	0.38 ± 0.11	− 0.405•	0.686	NS
	Range	0.1–0.59	0.17–0.59			
AR	0	12 (12.4%)	3 (6.5%)	3.036*	0.552	NS
	1	54 (55.7%)	32 (69.6%)			
	2	28 (28.8%)	10 (21.7%)			
	3	3 (3.1%)	1 (2.2%)			
	4	0 (0.0%)	0 (0.0%)			
RVSP (mmHg)	Mean ± SD	42.47 ± 10.96	41.04 ± 11.16	0.721•	0.472	NS
	Range	25–77	20–86			

CT						
Annulus. Diameter (mm)	Mean ± SD	24.49 ± 1.69	23.49 ± 1.63	3.344	0.001	HS
Annulus. Diameter.indexed (mm/m^2^)	Mean ± SD	13.13 ± 1.44	12.69 ± 1.27	1.774	0.078	NS
Annulus. Perimeter (mm)	Mean ± SD	79.06 ± 4.62	75.67 ± 5.31	3.910	0.000	HS
Annulus. Perimeter. Indexed (mm/m^2^)	Mean ± SD	42.38 ± 4.35	40.88 ± 4.14	1.954	0.053	NS
Annulus. Area (mm^2^)	Mean ± SD	474.14 ± 59.63	432.15 ± 60.36	3.890	0.000	HS
Annulus. Area indexed (mm^2^/m^2^)	Mean ± SD	253.93 ± 37.41	233.39 ± 33.69	3.137	0.002	HS
LMCA (mm)	Mean ± SD	12.89 ± 2.78	13.10 ± 2.24	− 0.448	0.655	NS
LMCAi (mm/m^2^)	Mean ± SD	6.91 ± 1.62	7.07 ± 1.26	− 0.578	0.564	NS
RCA (mm)	Mean ± SD	14.95 ± 3.26	14.95 ± 2.75	− 0.003	0.998	NS
RCAi (mm/m^2^)	Mean ± SD	8.04 ± 2.02	8.11 ± 1.77	− 0.207	0.837	NS
MS (mm)	Mean ± SD	8.91 ± 2.14	8.63 ± 1.86	0.765	0.445	NS
MS I (mm/m^2^)	Mean ± SD	4.80 ± 1.32	4.69 ± 1.18	0.453	0.651	NS
Grade of aortic calcification	Grade 1	0 (0.0%)	1 (2.2%)	9.820*	0.020	S
	Grade 2	17 (17.5%)	7 (15.2%)			
	Grade 3	33 (34.0%)	26 (56.5%)			
	Grade 4	47 (48.5%)	12 (26.1%)			
Severe or non severe aortic calcification	Grade 1–3	50 (51.5%)	34 (73.9%)	6.441*	0.011	S
	Grade 4	47 (48.5%)	12 (26.1%)			
Septal calcification	No	81 (83.5%)	42 (91.3%)	1.578*	0.209	NS
	Yes	16 (16.5%)	4 (8.7%)			

*AF* Atrial fibrillation, *AR* Aortic regurgitation, *AVA* Aortic valve area, *AVAi* Aortic valve area indexed, *BMI* Body mass index, *BSA* Body surface area, *Ca* Calcification, *CABG* Coronary artery bypass graft, *CLD* Chronic lung disease, *CrCl* Creatinine clearance, *CVS* Cerebrovascular stroke, *DM* Diabetes mellitus, *EF* Ejection fraction, *HTN* Hypertension, *IHD* Ischemic heart disease, *LVEDD* Left ventricle end diastolic dimension, *LVEDDi* Left ventricle end diastolic dimension indexed, *LVESD* Left ventricle end systolic dimension, *LVESDi* Left ventricle end systolic dimension indexed, *LMCA* Left main coronary artery height, *LMCAi* Left main coronary artery height indexed, *MPG* Mean pressure gradient, *MS* Membranous septum, *MSi* Membranous septum indexed, *PWT* Posterior wall thickness, *PWTi* Posterior wall thickness indexed, *PPG* Peak pressure gradient, *RCA* Right coronary artery height, *RCAi* Right coronary artery height indexed, *RHD* Rheumatic heart disease, *RVSP* Right ventricular systolic pressure, *STS PROM* Society of thoracic surgery predicted risk of mortality, *SWT* Septal wall thickness, *SWTi* Septal wall thickness indexed

*P*-value > 0.05: Non significant (NS); *P*-value <0.05: Significant (S); *P*-value< 0.01: highly significant (HS)

^‡^Mann Whitney test, *Chi-square test, •Independent t-test

The descriptive analysis of the demographic characteristics and clinical risk factors revealed no significant difference between both groups; however, the Euro score II and STS scores were relatively higher in group A, with median values of 3.56 and 2.58, respectively. (*P* value = 0.01 and 0.03 respectively).The ECG and echocardiographic measurement data also revealed no difference between both groups.

The CT data measurement confirmed the relatively smaller aortic annulus perimeters in group B 75.67 ± 5.31 mm vs 79.06 ± 4.62 mm in group A. (*P* value = 0.000). The aortic cusps and annulus calcification grades were relatively higher in group A. Severe grades of calcification represented 48.9% of patients in group A vs. 26.1% of patients in group B (*P* value = 0.011).There was no clinically significant difference between both groups in other CT measurement data, like the heights of coronary arteries from the aortic annulus and membranous septum length.

### Comparison of operative and post operative data between group A and group B. (Table 5)

**Table 5:** Comparison between group A and extreme oversizing group B regarding operative and post operative data

		Group A (< 20%)		Group B (> = 20%)		Test value	P-value	Sig.
		No.	%	No.	%			
THV size	29.00	78	80.4%	31	67.4%	2.919*	0.088	NS
	34.00	19	19.6%	15	32.6%			

Operative data		Group A (< 20%)	Group B (≥ 20%)		Test value	*P*-value	Sig.	
		No. = 97	No. = 46					
DI (mm)	Median (IQR)	3.3 (2.7–5.2)	2.55 (1.6–3.7)		− 3.560‡	0.000	HS	
	Range	1–8	1–8					
DI I (mm/m^2^)	Median (IQR)	1.81 (1.44–2.75)	1.47 (0.82–1.88)		− 3.468‡	0.001	HS	
	Range	0.47–4.6	0.42–4.42					
Pre- implantation dilatation	No	73 (75.3%)	34 (73.9%)		0.030*	0.863	NS	
	Yes	24 (24.7%)	12 (26.1%)					
Post- implantation dilatation	No	80 (82.5%)	38 (82.6%)		0.000*	0.984	NS	
	Yes	17 (17.5%)	8 (17.4%)					
DIMS%	Median (IQR)	39.53 (28.39–61.39)	28.17 (18.89–45.54)		− 3.269	0.001	HS	
	Range	10–114.23	10–76.19					

ECG post operative		Group A (< 20%)		Group B (≥ 20%)		Test value*	*P*-value	Sig.
		No.	%	No.	%			
Conduction Disturbance	No	74	76.3%	39	84.8%	1.358	0.2438	NS
	Yes	23	23.7%	7	15.2%			
Permanent CHB	No	95	97.9%	46	100.0%	0.962	0.327	NS
	Yes	2	2.1%	0	0.0%			
Transient Conduction. Disturbance	No	89	91.8%	43	93.5%	0.131	0.718	NS
	Yes	8	8.2%	3	6.5%			
New LBBB	No	79	81.4%	40	87.0%	0.679	0.410	NS
	Yes	18	18.6%	6	13.0%			
QRS widening	No	75	77.3%	39	84.8%	1.075	0.300	NS
	Yes	22	22.7%	7	15.2%			
PR Prolongation	No	91	93.8%	46	100.0%	2.970	0.085	NS
	Yes	6	6.2%	0	0.0%			
New onset AF	No	95	97.9%	45	97.8%	0.002	0.965	NS
	Yes	2	2.1%	1	2.2%			

Postoperative data		Group A (< 20%)	Group B (≥ 20%)	Test value	*P*-value	Sig.
		No. = 97	No. = 46			
PVL	No (0)	22 (22.7%)	39 (84.8%)	49.761*	0.000	HS
	Trivial (1)	61 (62.9%)	7 (15.2%)			
	Mild (2)	12 (12.4%)	0 (0.0%)			
	Moderate(3)	2 (2.1%)	0 (0.0%)			
	Severe (4)	0 (0.0%)	0 (0.0%)			
PVL	Non significnat	83 (85.6%)	46 (100.0%)	7.360*	0.007	HS
	Significant 2–4	14 (14.4%)	0 (0.0%)			
Coronary obstruction	No	97 (100.0%)	46 (100.0%)	NA	NA	NA

*CHB* Complete heart block, *DIDO* Depth of implantation derived oversizing index, *DI* Depth of implantation, *DI* Depth of implantation indexed, *DIMS* Depth of implantation to membranous septum ratio, *LBBB* Left bundle branch block, *PVR* Para-valvular leakage, *THV* Trans catheter heart valve

P-value > 0.05: Non significant (NS); P-value < 0.05: Significant (S); P-value < 0.01: highly significant (HS)

^‡^Mann Whitney test

*Chi-square test

•Independent *t*-test

The (Evolut R) Valve size 29 was implanted in 109 patients (76.2%) in the whole population, with 78 patients (80.4%) belonging to group A and 31 patients (67.4%) belonging to group B. The( Evolut R) Valve size 34 was implanted in 34 patients (23.8%) in the whole population, with 19 patients (19.6%) belonging to group A and 15 patients (32.6%) belonging to group B. There was no significant difference between both groups in the concept of the implanted valve size. )*P* value = 0.08).

The operative statistics revealed that the implanted valves in group A were relatively deeper than those in group B, with higher DI, DIi, and DIMS indexes. There was no statistically significant difference between both groups in terms of new-onset conduction disturbance, with an incidence of 23.7% in group A vs. 15.2% in group B. However, the two cases that required a permanent pacemaker belonged to group A.There was more clinically significant PVL in group A than in group B (*P* value 0.007).

### Comparison between populations with no NOCD and populations with NOCD (Table 6)

**Table 6:** Showing Comparison between populations without NOCD and populations with NOCD

		No conduction dist	Conduction dist	Test value	P-value	Sig
		No. = 113	No. = 30			
Age (years)	Female	49 (43.4%)	11 (36.7%)	0.436*	0.509	NS
	Male	64 (56.6%)	19 (63.3%)			
Weight (kg)	Mean ± SD	73.75 ± 6.59	74.00 ± 6.53	− 0.183•	0.855	NS
	Range	55–88	60–83			
Height (m)	Mean ± SD	79.49 ± 14.62	78.60 ± 13.55	0.301•	0.764	NS
	Range	50–125	50–120			
BMI(kg/m^2^)	Mean ± SD	1.64 ± 0.07	1.66 ± 0.08	− 1.243•	0.216	NS
	Range	1.5–1.82	1.5–1.82			
BSA (m^2^)	Mean ± SD	29.28 ± 5.45	28.27 ± 3.94	0.949•	0.344	NS
	Range	18.82–47.87	22.22–41.52			
Age (years)	Mean ± SD	1.87 ± 0.18	1.89 ± 0.18	− 0.626•	0.532	NS
	Range	1.52–2.39	1.44–2.38			
Euro score II %	Median (IQR)	3.35 (1.85–4.56)	4.67 (2.3–7.91)	− 2.358‡	0.018	S
	Range	0.84–40.2	0.91–13.98			
STS score %	Median (IQR)	2.4 (1.75–3.2)	3.37 (1.57–4.56)	− 2.066‡	0.039	S
	Range	0.66–16.98	0.76–5.99			
CABG	No	103 (91.2%)	27 (90.0%)	0.038*	0.846	NS
	Yes	10 (8.8%)	3 (10.0%)			
Smoking	Non-smoker	87 (77.0%)	20 (66.7%)	4.327*	0.115	NS
	Smoker	20 (17.7%)	5 (16.7%)			
	EX -smoker	6 (5.3%)	5 (16.7%)			
DM	No	63 (55.8%)	12 (40.0%)	2.359*	0.125	NS
	Yes	50 (44.2%)	18 (60.0%)			
HTN	No	26 (23.0%)	7 (23.3%)	0.001*	0.970	NS
	Yes	87 (77.0%)	23 (76.7%)			
IHD	No	75 (66.4%)	12 (40.0%)	6.920*	0.009	HS
	Yes	38 (33.6%)	18 (60.0%)			
CVS	No	109 (96.5%)	25 (83.3%)	6.926*	0.008	HS
	Yes	4 (3.5%)	5 (16.7%)			
CrCl (ml/mm^2^)	Mean ± SD	64.38 ± 27.42	60.00 ± 24.09	0.796•	0.427	NS
	Range	9–161	12–117			
CLD	No	105 (92.9%)	27 (90.0%)	0.285*	0.594	NS
	Yes	8 (7.1%)	3 (10.0%)			
Grade of aortic Calcification	Grade 1	0 (0.0%)	1 (3.3%)	5.091*	0.165	NS
	Grade 2	21 (18.6%)	3 (10.0%)			
	Grade 3	47 (41.6%)	12 (40.0%)			
	Grade 4	45 (39.8%)	14 (46.7%)			
Severe or non severe aortic Calcification	Grade 1–3	68 (60.2%)	16 (53.3%)	0.458*	0.499	NS
	Grade 4	45 (39.8%)	14 (46.7%)			
Septal Calcification	No	101 (89.4%)	22 (73.3%)	5.075*	0.024	S
	Yes	12 (10.6%)	8 (26.7%)			
Annulus. Diameter.indexed (mm/m2)	Mean ± SD	24.08 ± 1.71	24.50 ± 1.78	− 1.172	0.243	NS
	Range	20–28.2	22–28.4			
Annulus. Perimeter (mm)	Mean ± SD	12.98 ± 1.45	13.01 ± 1.18	− 0.120	0.905	NS
	Range	8.93–17.48	10.71–17.01			
Annulus. Perimeter. Indexed (mm/m^2^)	Mean ± SD	77.70 ± 4.93	78.97 ± 5.62	− 1.210	0.228	NS
	Range	69–90.1	70–92			
Annulus. Area (mm2)	Mean ± SD	41.87 ± 4.42	41.98 ± 4.02	− 0.121	0.904	NS
	Range	34.17–56.6	34.45–52.08			
Annulus. Area indexed (mm^2^/m^2^)	Mean ± SD	457.71 ± 59.77	472.49 ± 72.90	− 1.146	0.254	NS
	Range	339–615	379–666			
Annulus. Diameter.indexed (mm/m^2^)	Mean ± SD	246.59 ± 37.62	250.50 ± 37.03	− 0.507	0.613	NS
	Range	166.91–377	200.87–348.69			
DI (mm)	Median (IQR)	3 (2.2–3.7)	5.35 (3.5–7.22)	− 3.560‡	0.000	HS
	Range	1–7.19	1.3–8			
DI I (mm/m^2^)	Median (IQR)	1.59 (1.16–1.96)	2.71 (1.92–3.68)	− 3.468‡	0.001	HS
	Range	0.42–4.05	0.76–4.6			
DIMS %	Median (IQR)	33.72 (23.96–45.88)	69.26 (31.82–83.6)	− 3.269‡	0.001	HS
	Range	10–93.38	10–114.23			
Pre implantation-Dilatation	No	84 (74.3%)	23 (76.7%)	0.068*	0.794	NS
	Yes	29 (25.7%)	7 (23.3%)			
Post- implantation Dilatation	No	95 (84.1%)	23 (76.7%)	0.901*	0.343	NS
	Yes	18 (15.9%)	7 (23.3%)			
RHD	No	110 (97.3%)	26 (86.7%)	5.807*	0.016	S
	Yes	3 (2.7%)	4 (13.3%)			
MS(mm)	Mean ± SD	8.76 ± 1.95	9.07 ± 2.43	− 0.749•	0.455	NS
	Range	5–14.4	5.3–15.3			
MS i (mm/m^2^)	Mean ± SD	4.74 ± 1.21	4.87 ± 1.52	− 0.500•	0.618	NS
	Range	2.58–8.82	2.72–8.06			
DIDO %	Mean ± SD	17.23 ± 5.79	13.98 ± 5.86	2.723•	0.007	HS
	Range	0–30	1–23			

*BMI* Body mass index, *BSA* Body surface area, *Ca* Calcification, *CABG* Coronary artery bypass graft, *CLD* Chronic lung disease, *CrCl* Creatinine clearance, *CVS* Cerebrovascular stroke, *DIDO* Depth of implantation derived oversizing index, *DI* Depth of implantation, *DI* Depth of implantation indexed, *DIMS* Depth of implantation to membranous septum ratio, *DM* Diabetes mellitus, *EF* Ejection fraction, *HTN* Hypertension, *IHD* ischemic heart disease, *MS* Membranous septum, *MSi* Membranous septum indexed, *RHD* Rheumatic heart disease, *STS PROM* Society of thoracic surgery predicted risk of mortality

*P*-value > 0.05: Non significant (NS); *P*-value < 0.05: Significant (S); *P*-value < 0.01: highly significant (HS)

^‡^Mann Whitney test *:Chi-square test; •: Independent t-test

**Fig. 9:**
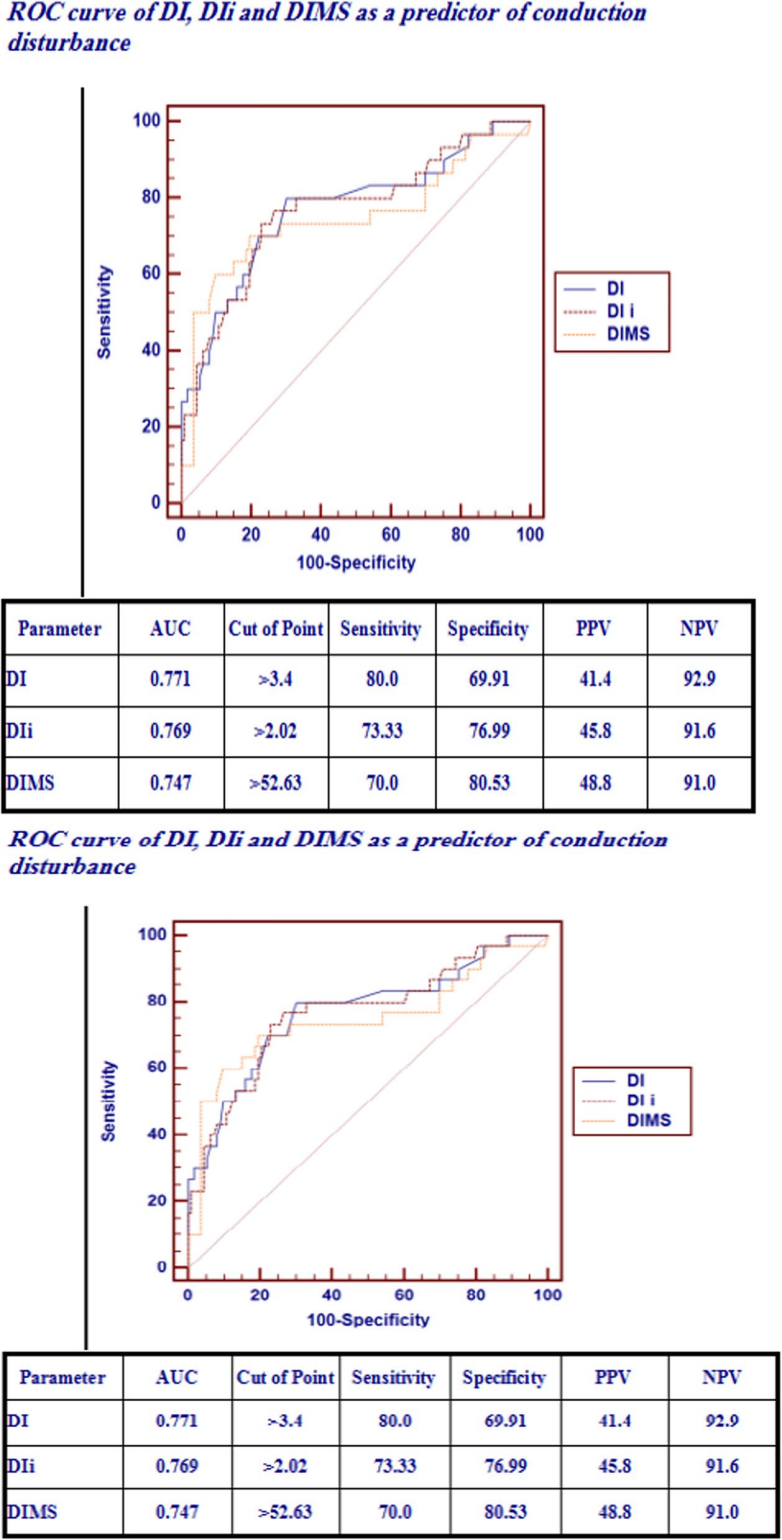
The COVs of Euro scores II and STS scores,DI,DIi and DIMS significantly associated with NOCD using ROC curves

The statistical analysis using the Chi-square test, independent *t*-test, Mann–Whitney test, logistic regression analysis, and ROC curves (as shown in Fig. 9) for detecting the cut-off values (COVs) has found that many factors can be associated with or predisposing to conduction disturbance, which included (Table 7):

1. Euro score II > 5.58, STS score > 3.23, Septal Ca, and RHD (*P*-value** < 0.05)**
2. IHD, CVS, DI > 3.4 mm, DI i > 2.02 mm/m^2^, and DIMS more than 52.63% (P-value < 0.01).
3. DIDO was inversely related to NOCD.
4. By doing multivariate analysis, we found that IHD and RHD are the two most predictive variants from a statistical point of view.
5. The main results of our study concerning the predictors of conduction disturbance did not include the depth of implantation-derived oversizing (DIDO) as a predictor.

**Table 7:** Logistic regression analysis for predictors of conduction disturbance

	Uni-variate				Multi-variate			
	P-value	Odds ratio (OR)	95% C.I. for OR		P-value	Odds ratio (OR)	95% C.I. for OR	
			Lower	Upper			Lower	Upper
Euro score II > 5.58	0.001	4.069	1.714	9.661	0.711	0.735	0.145	3.739
STS score > 3.23	0.001	4.023	1.730	9.353	0.086	3.556	0.835	15.144
IHD	0.010	2.961	1.293	6.777	0.042	3.176	1.045	9.650
CVS	0.016	5.450	1.365	21.767	0.642	1.601	0.221	11.601
Septal Ca	0.029	3.061	1.119	8.373	0.269	2.407	0.506	11.440
DI > 3.4(mm)	0.000	9.294	3.485	24.783	0.389	2.454	0.319	18.903
DI i > 2.02 (mm/m2)	0.000	8.759	3.500	21.924	0.558	1.656	0.306	8.960
DIMS > 52.63%	0.000	9.652	3.888	23.957	0.181	2.947	0.604	14.374
RHD	0.029	5.641	1.189	26.758	0.004	32.239	3.044	341.432
DIDO ≤ 21%	0.024	10.482	1.367	80.349	0.176	5.228	0.476	57.425

*CVS* Cerebrovascular stroke, *DIDO* Depth of implantation derived oversizing index, *DI* Depth of implantation, *DI* Depth of implantation indexed, *DIMS* Depth of implantation to membranous septum ratio, *IHD* Ischemic heart disease, *RHD*: Rheumatic heart disease, Society of thoracic surgery predicted risk of mortality

### Comparison between population with no significant PVL and population with significant PVL (Table 8)

**Table 8:** Showing comparison between population without significant PVL and population with significant PVL

		Non significant PVL	Significant PVL	Test value	*P*-value	Sig.
		No. = 129	No. = 14			
Age (years)	Female	54 (41.9%)	6 (42.9%)	0.005*	0.943	NS
	Male	75 (58.1%)	8 (57.1%)			
Weight (kg)	Mean ± SD	73.82 ± 6.23	73.64 ± 9.33	0.097•	0.923	NS
	Range	59–87	55–88			
Height (m)	Mean ± SD	79.00 ± 14.39	82.07 ± 14.33	− 0.758•	0.450	NS
	Range	50–125	50–99.5			
BMI(kg/m^2^)	Mean ± SD	1.65 ± 0.07	1.64 ± 0.09	0.227•	0.820	NS
	Range	1.5–1.82	1.5–1.82			
BSA (m^2^)	Mean ± SD	29.07 ± 5.19	29.09 ± 5.22	− 0.010•	0.992	NS
	Range	18.82–47.87	22.22–41.42			
Age (years)	Mean ± SD	1.87 ± 0.17	1.88 ± 0.21	− 0.083•	0.934	NS
	Range	1.52–2.39	1.44–2.1			
Euro score II %	Median (IQR)	3.35 (1.95–5.2)	4.26 (2.56–10.1)	− 1.654‡	0.098	NS
	Range	0.84–40.2	1.24–14.54			
STS score %	Median (IQR)	2.45 (1.7–3.52)	2.97 (1.89–3.78)	− 0.988‡	0.323	NS
	Range	0.66–16.98	1.27–10.2			
CABG	No	117 (90.7%)	13 (92.9%)	0.071*	0.790	NS
	Yes	12 (9.3%)	1 (7.1%)			
Smoking	Non-smoker	97 (75.2%)	10 (71.4%)	0.992*	0.609	NS
	Smoker	23 (17.8%)	2 (14.3%)			
	EX -smoker	9 (7.0%)	2 (14.3%)			
DM	No	64 (49.6%)	11 (78.6%)	4.247*	0.039	S
	Yes	65 (50.4%)	3 (21.4%)			
HTN	No	29 (22.5%)	4 (28.6%)	0.264*	0.607	NS
	Yes	100 (77.5%)	10 (71.4%)			
IHD	No	78 (60.5%)	9 (64.3%)	0.077*	0.781	NS
	Yes	51 (39.5%)	5 (35.7%)			
CVS	No	121 (93.8%)	13 (92.9%)	0.019*	0.890	NS
	Yes	8 (6.2%)	1 (7.1%)			
CrCl (ml/m^2^)	Mean ± SD	63.70 ± 27.15	61.23 ± 23.30	0.327•	0.744	NS
	Range	9–161	24.32–106			
CLD	No	119 (92.2%)	13 (92.9%)	0.007*	0.935	NS
	Yes	10 (7.8%)	1 (7.1%)			
Severe or non severe aortic Calcification	Grade (1–3)	80 (62.0%)	4 (28.6%)	5.829*	0.016	S
	Grade 4	49 (38.0%)	10 (71.4%)			
Grades of aortic calcifications	Grade 1	1 (0.8%)	0 (0.0%)	6.742*	0.081	NS
	Grade 2	24 (18.6%)	0 (0.0%)			
	Grade 3	55 (42.6%)	4 (28.6%)			
	Grade 4	49 (38.0%)	10 (71.4%)			
Septal Calcification	No	111 (86.0%)	12 (85.7%)	0.001*	0.973	NS
	Yes	18 (14.0%)	2 (14.3%)			
Annulus diameter indexed (mm/m^2^)	Mean ± SD	24.14 ± 1.75	24.43 ± 1.53	− 0.596•	0.552	NS
	Range	20–28.4	22.6–27.8			
Annulus perimeter (mm)	Mean ± SD	12.97 ± 1.32	13.18 ± 1.99	− 0.557•	0.579	NS
	Range	8.93–16.99	11.21–17.48			
Annulus perimeter. Indexed (mm/m^2^)	Mean ± SD	77.88 ± 5.12	78.77 ± 4.85	− 0.615•	0.539	NS
	Range	69–92	73.2–90.1			
Annulus Area (mm^2^)	Mean ± SD	41.83 ± 4.13	42.46 ± 6.02	− 0.517•	0.606	NS
	Range	34.17–53.78	36.7–56.6			
Annulus Area indexed (mm^2^/m^2^)	Mean ± SD	459.61 ± 62.92	471.99 ± 62.72	− 0.699•	0.486	NS
	Range	339–666	392.6–601			
Annulus Diameter.indexed (mm/m^2^)	Mean ± SD	246.62 ± 36.06	254.71 ± 49.07	− 0.768•	0.444	NS
	Range	166.91–355.3	203.1–377			
DI (mm)	Median (IQR)	3.15 (2.4–4.6)	2.8 (2–5.2)	− 0.347‡	0.729	NS
	Range	1–8	1–8			
DI I (mm/m^2^)	Median (IQR)	1.74 (1.27–2.41)	1.57 (1.19–2.64)	− 0.071‡	0.943	NS
	Range	0.42–4.6	0.51–4.24			
DIMS %	Median (IQR)	37.5 (24.74–58)	29.25 (24.1–62.5)	− 0.455‡	0.649	NS
	Range	10–113.9	14.29–114.23			
Pre implantation-dilatation	No	99 (76.7%)	8 (57.1%)	2.576*	0.108	NS
	Yes	30 (23.3%)	6 (42.9%)			
Post- implantation dilatation	No	117 (90.7%)	1 (7.1%)	61.119*	0.000	HS
	Yes	12 (9.3%)	13 (92.9%)			
RHD	No	122 (94.6%)	14 (100.0%)	0.799*	0.371	NS
	Yes	7 (5.4%)	0 (0.0%)			
DIDO%	Mean ± SD	16.94 ± 5.91	12.93 ± 4.98	2.445•	0.016	S
	Range	1–30	0–19			

*BMI* Body mass index, *BSA* Body surface area, *Ca* Calcification, *CABG* Coronary artery bypass graft, *CLD* Chronic lung disease, *CrCl* Creatinine clearance, *CVS* Cerebrovascular stroke, *DIDO* Depth of implantation derived oversizing index, *DI* Depth of implantation, *DI* Depth of implantation indexed, *DIMS* Depth of implantation to membranous septum ratio, *DM* Diabetes mellitus, *EF* Ejection fraction, *HTN* Hypertension, *IHD* ischemic heart disease, *MS* Membranous septum, *MSi* Membranous septum indexed, *RHD* Rheumatic heart disease, *STS PROM* Society of thoracic surgery predicted risk of mortality

*P*-value > 0.05: Non significant (NS); *P*-value < 0.05: Significant (S); *P*-value < 0.01: highly significant (HS)

^‡^Mann Whitney test

*Chi-square test

•Independent *t*-test

**Fig. 10:**
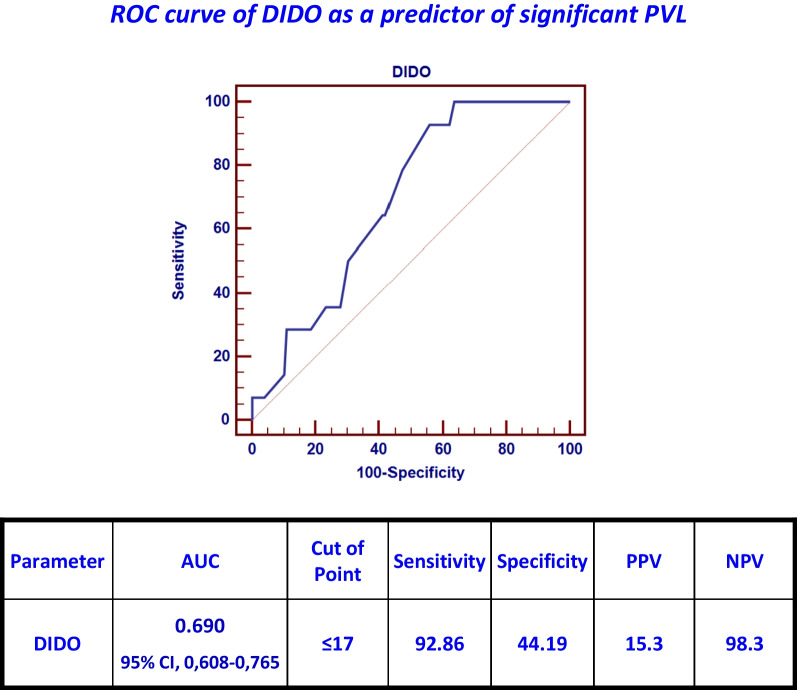
ROC curve of DIDO as a predictor of significant PVL

In regards to significant PVL (more than or equal to grade 2); Diabetes mellitus, the depth of implantation-derived oversizing index (DIDO) of less than 17%, and only severe degrees of aortic valve calcification (grade 4) were clinically and statistically important predictors. The cutoff point of the depth of implantation derived oversizing index (DIDO) to predict significant PVL was less than 17% using the ROC curve with relatively higher specificity (44.19%) and AUC, 0.69; 95% CI, 0.608–0.765 (Fig. 10).

The logistic regression analysis has excluded diabetes mellitus as an independent clinical predictor for significant PVL.The other two clinical and statistical factors that can predict clinically significant PVL were analyzed using multivariate regression. DIDO was the most important independent factor to predict significant PVL with an OR of 8.704 and a CI of (1.091–69.459) (Table 9).

**Table 9:** Logistic regression analysis for predictors of significant PVL

	Uni-variate				Multi-variate			
	*P*-value	Odds ratio (OR)	95% C.I. for OR		*P*-value	Odds ratio (OR)	95% C.I. for OR	
			Lower	Upper			Lower	Upper
DM	0.051	0.269	0.072	1.008	–	–	–	–
(grade4 aortic valve calcification)	0.023	4.082	1.214	13.726	0.056	3.345	0.970	11.543
DIDO ≤ 17%	0.027	10.292	1.307	81.025	0.041	8.704	1.091	69.459

*DM* Diabetes mellitus, *DIDO* Depth of implantation derived oversizing index

## Discussion

TAVI has become the first line of treatment for the elderly population with severe symptomatic aortic stenosis, especially those with high or moderate operative risk. In the current era, the choice of percutaneous intervention has gained a higher level of evidence, even in low-risk individuals [2].

One of the main complications that can still affect the clinical outcomes of the procedure is para-valvular leakage (PVL), even if it is present in mild grades as it is associated with higher all-cause mortality and re-hospitalization [5, 6, 20].

The major predictors of the PVL can be classified into procedural factors and anatomical characteristics [20]. Anatomical factors include the degree of device landing zone calcium, and LVOT eccentricity [20, 24].

The procedural factors are related to the choice of the type of device, whether self-expandable (SE) or balloon-expandable (BE), with more radial force related to the balloon-expandable valve [25], Accordingly in cases of mild to moderate calcification, both types can perform equally, unlike cases of higher grades of calcification, BE valves can be preferred to decrease the expected PVL, especially if the calcification does not reach out to the annulus. The other device-related factor is the use of early versus newer generations that have leak-proof functions (covered by outer sealing skirts). Those devices include, but are not limited to, Evolut R PRO and SAPIEN 3 [26].

Furthermore, the incidence of PVL is related to the positioning of the valve and the oversizing index (OI), which is beneficial mainly to valves without leak proof function like Evolut R valves. Most of the recent studies recommend an OI of 14 to 17% and around 15% for valves with leak proof function as the upper limit [11, 20]. There is scarce data available about oversizing of 20% or more and its impact when using valves without leak-proof functions [10].

Based on the previous data,PVL can be reduced by pre-procedurally identifying the anatomical features and choosing the most suitable available device as well as performing the best degree of over sizing index (OI) according to the type of the device and the anatomical characteristic.

In developing countries with limited resources the luxury of choosing a certain valve type to suit a specific patient may not be present. The available valve during the study period in our cardiac catheterization -labs was Evolut R, so we had to devise techniques for its use in order to limit complications.

The main interest of our study focused on detecting the most accurate oversizing index with the best results and the least risk of PVL and annular rupture.

To the best of our knowledge, the data provided by the manufacturer that regarding the degree of oversizing for every millimeter of the depth of implantation has not been studied before.

This study is the first to integrate new data for calculating the oversizing index (OI) in the case of Evolut R devices based on the depth of implantation. Our study aimed not only to identify the effect of oversizing based on the depth of implantation as a modifier of PVL risk, but also to propose a predictive model which might help modify of the implantation technique aiming at reducing the incidence of Paravalvular leakage without inducing conduction disturbance complicating TAVI.

The Evolut R valve is a bulky valve with a 45 mm length that needs a large area of implantation. Moreover, being a SE valve, it has less radial force than (BE) valves. Both features have advantages and disadvantages [27].However, the lower radial force of Evolut R devices in comparison with (BE) valves makes it safer in the setting of annular calcification [27, 28]. Its efficacy to prevent PVL in the absence of the optimum degree of oversizing and in the presence of high grades of calcium is less than that of (BE) valves [29]. The large device size allows easy implantation, but it can affect the conduction system, or the device can be affected by the LVOT calcification.

In our study, patients with severe LVOT calcification were excluded due to possible contribution to significant PVL even with performing the optimum degree of oversizing [24, 30] Patients with preexisting conduction disturbance were excluded as well in order to detect the isolated effect of the oversizing on the conduction system.

In our study, group B (oversizing of 20% or more) had a mild to moderate aortic valve calcification prevalence of 73.9% versus 51.5% in group A, while severe calcification was more prevalent in group A (48.5% vs. 26.1% in group B), as we tend to avoid oversizing in cases of severe aortic valve calcification (AVC). This goes in line with Barbanti et al., who recommended avoiding oversizing in severe AVC to avoid annular rupture [27].

The main findings of the study were consistent with our study interests. We found that oversizing by 20% or more had no hazardous effect on the coronary blood flow or the annular integrity and better outcomes concerning the degree of the post-procedural PVL. We found new predictors of new onset conduction disturbances (NOCD) other than the preexisting conduction abnormalities. The depth of implantation-derived oversizing index (DIDO) was inversely related to the incidence of new-onset conduction disturbance (NOCD), indicating that DIDO is not a predisposing factor for NOCD.

Only two cases required permanent pacemaker (PPM) implantation. Both cases had been implanted with devices of size 34 with an oversizing index of 8%. In the first case [patient no. 35], NOCD could be attributed to deep implantation (8 mm), while in the second case [patient no. 127], grade 4 aortic valve calcification and ischemic heart disease were the major contributors. The previous data confirm once again that oversizing is not an independent predictor of NOCD.

According to the study results, oversizing by at least 17% can reduce significant PVL in absence of severe LVOT calcification. In this study, two cases ended up with grade 3 (moderate) PVL. One of those cases had zero percent oversizing [patient no. 7], and the other case had a 17% oversizing index but grade 4 aortic calcification [patient no. 119]. This boosts the fact that oversizing cannot always overcome anatomical factors, Therefore avoiding extreme oversizing is a sound practice in cases of severe annular calcification in terms of safety and without missing a lot of added benefits.

Drakopoulou et al. [11] have proposed oversizing by 14% as a cutoff point to reduce significant PVL with the least incidence of PPM using ROC analysis in a retrospective pattern (AUC, 0.806; 95% confidence interval [CI], 0.706–0.905; *P* < 0.01). The reported incidence of significant PVL was 19% in group 1 (oversizing by less than 14%) vs. 3% in the other group *P* < 0.01.In our study, less than 17% of oversizing was predictive of significant PVL by similar analysis (AUC, 0.69; 95% CI, 0,608–0,765; *P* = 0.04).Similarly, our study reported a higher incidence of significant PVL in group A (oversizing by less than 20%) than in group B (14.4% vs. 0%, respectively, and a P value of 0.007). The differences in the reported rates of PVL may be attributed to different study populations and different degrees of oversizing.

Our study conform with Ki et al. [20] who have set 17.3% or more as the optimum OI for predicting no or trace PVL in valves without leak-proof function (AUC, 0.639, *P* = 0.018) and an upper limit of OI of 30%. Moreover there was no significant difference in OI regarding PPM insertion (PPM insertion vs. no insertion, OI, 14.2 ± 10.8% vs. 13.7 ± 7.0%, *P* = 0.893). There was no difference in the volume of calcium regarding PPM insertion (PPM insertion vs. no insertion, 351 mm3 vs. 551 mm3, *P* = 0.776). The relatively similar Cov of OI predicting non-significant PVL can be explained by close population size since their study included 37 CoreValves 56 Evolut R valves,and 19 Evolut PRO valves.Similarly in our study, new-onset conduction disturbance (NOCD) was not related to the grade of aortic valve calcification, That's to say, there was no significant difference between the population without NOCD and the population with NOCD as regards grade 4 aortic valve calcification (38% vs. 46.7%, *P* = 0.45). Furthermore, NOCD was not related to the degree of OI. There was no significant difference between group A and group B in our study as regards NOCD (23.7% vs. 15.2%, respectively, *P* value 0.244) which apparently nullifies the effect of oversizing on NOCD.

Likewise, our study results were in accordance with the study by Ammar, et al. [10] which detected that the incidence rate of significant PVL decreased from 7.4 to 2.8% when comparing OI by less than 20% vs. more than 20% (*P* value = 0.02). Over and above, there was no significant difference as regards the rates of PPM implantation in the population with OI less than 20% vs. the population with OI more than 20% (13 vs. 12%, p value more than 0.05). Similarly, our study noted that the rates of significant PVL decreased from 14.4 to 0% when comparing the population with OI less than 20% vs. those with OI more than 20% (*P* value = 0.007). In our study, there was no significant difference as regards the rates of PPM implantation among both groups (2.1% vs. 0%, *P* value = 0.327), and the cases that required PPM were belonged to group A. The difference in the incidence rates as regards the PVL or PPM between both studies might be explained by different population size.

Our study opposed Mauri V. et al.’s study [29] (*n* = 212 Accurate Neo), which concluded that there was no association between significant PVL and the oversizing index (*P* = 0.259) or eccentricity of the aortic annulus (*P* = 0.062), and that aortic valve calcification (AVC) more than 410.6 mm3 (OR 6.9, CI 3.0–15.8; *P* 0.001) was solely the independent predictor of significant PVL after multivariate regression analysis. On the contrary, our study found that only OI less than 17% is the independent predictor of significant PVL after multivariate analysis with OR 8.7 and *P* value = 0.04. However, the wide range of CI in the multivariate analysis can be explained by the Presence of only single case with significant PVL in population with OI more than 17%. The different conclusions may be due to different (SE) valve types.

In contrast to Hagar, et al.’s study [31] (*n* = 270,different types of valves) suggesting that AVC and the larger annular perimeters and areas were significantly associated with significant PVL, our study found that there was no significant association between the annulus area or perimeter even when indexed to BSA and significant PVL (*P* values 0.44, 0.60, respectively). However, meaningful comparisons cannot be warranted due to different methodologies and different population sizes.

Our study could also prove that, apart from the size of the valve, the oversizing index is affected by the depth of implantation. Deeper implantation was observed in group A (oversizing less than 20%) than in group B (oversizing equal to or more than 20%), with a *P* value of 0.001. To the best of our knowledge, such a parameter was not studied before and further studies are needed for verification.

Other than the preexisting conduction disturbances, new predictors for NOCD were depicted using uni-variate logistic regression. These predictors are RHD, Euro score II more than 5.58, STS score more than 3.28, septal Calcification, IHD, CVS, DI more than 3.4 mm, DI i more than 2.02 mm/m^2^, and DIMS more than 52.63%. By performing multivariate logistic regression analysis, IHD and RHD were found to be significant predictors, a hypothesis that needs further confirmation in larger studies.

According to Choi, et al. and Putra et al. [32, 33], cardiac magnetic resonance imaging (CMRI) in patients with RHD detected myocardial fibrosis surrounding the involved valve annulus and even in other parts of the myocardium. This evidence of myocardial fibrosis may be the predisposing factor for NOCD in our study, especially with cardiac interventions that may affect the conduction system. In our research, all cases with RHD who developed NOCD had new-onset LBBB.

The previous data can be explained in the setting of selecting patients with absent preexisting conduction disturbances,targeting the least depth of implantation, and avoiding extreme oversizing in the setting of severe aortic valve calcification and short membranous septum.

Boonyakiatwattana et al. [34] found that there was no correlation between perimeter-based oversizing and NOCD (*P* value = 0.338), a finding that is similar to our study. In our study,oversizing more than 20% was safe in the context of the NOCD,coronary encroachment even in smaller annular perimeters, as evidenced by our results, which revealed that group B had smaller annular perimeters (75.67 ± 5.31 mm versus 79.06 ± 4.62 in group A). However, we did not find that membranous septum(MS) is an independent predictor factor, as in their study. The last finding in our study may be attributed to avoiding deep implantation in case of short MS and non randomization of study population.

In contrast to Dallan et al. [35] who found that the safe and effective cut-off point for oversizing in those with annulus diameters less than 30 mm is 12% or more, our study found that the least effective cut-off point for oversizing effectively reducing significant PVL with no other hazards is 17%.This can be attributed to different study populations with larger aortic areas and perimeters in their study.

Our study goes in line with Majeed et al. [36] who found that there was no statistically significant difference between the rate of permanent pacemaker implantation in the minimally oversizing group (8% versus 13% in the severe or moderately oversizing group) (*P* value = 0.4).

Finally we concluded that oversizing by at least 20% is a safe and effective method of reducing significant PVL in our cohort of patients and any other similar populations. The upper limit in our study was 30% but it needs further verification to be considered as a safe upper limit value.

The concept of the-depth-of-implantation-derived oversizing is a more accurate method of calculating the oversizing index (OI).

The prosthesis oversizing was not a predictor of new onset conduction disturbance (NOCD) in our study. New predictors of new onset conduction disturbance in our study have been suggested but these predictors need more verification in larger trials.

## Conclusions

Prosthesis oversizing by 20% using the self-expandable Evolut R valve is safe and effective, with apparently no significant effect on the conduction system, coronary encroachment, or annular injury, and warrants a greater reduction in the incidence of significant PVL in any similar population to our study population.

### Recommendations

In the setting of absent severe LVOT calcification or annular calcification, performing depth of implantation-derived oversizing (DIDO) by less than 17% can predict a significant PVL degree, and the optimum oversizing should be 20%.

The depth of implantation can affect the degree of oversizing, especially in larger valve sizes, so it's a more accurate method to consider the depth of implantation during oversizing estimation. The deeper the implantation, the less than expected the degree of oversizing.

A larger population study with randomization is needed to verify these results as regards the effect of DIDO on NOCD and the independent predictors of significant PVL.

Further research is needed to verify the upper limit of OI and compare the traditional method of calculating the oversizing vs the DIDO method in a larger trial.

### Limitations

The study was performed on one type of SE valve (Evolut R) and on specific sizes of that type: 29 and 34. As regards the device type, the limitation was related to logistical regulation and device availability in our hospitals. As regards the device sizes, the limitation was related to the unavailability of their demos for performing the in vitro measurements. Another limitation was the sample size, which was relatively small, especially in the context of the numbers of cases with new-onset conduction disturbances or significant PVL, so results concerning their independent predictors need to be verified in a larger population size. The study was not randomized, and it was conducted in the context of the experience of the operators and also the pre-TAVI MDCT protocol. These factors could have prevented many hazards.

The study had been conducted in a specific setting to evaluate our primary end point regarding the safety and efficacy of the optimum degree of oversizing in order to avoid any effect by other confounding factors. In other circumstances, the predictors of NOCD and significant PVL could have been changed.

The manual measurement of the outer rim can be more accurate if it is done using LASER measurement, as our measurement of the outer diameters at different heights from its base, which is equivalent to the depth of implantation, was done at every 1 mm. As a result, we rounded decimal numbers of the implantation depth to the nearest integer, so we recommend verifying this experiment using LASER measurements on the same valve sizes and other sizes for more accuracy of the same concept, which is the depth of implantation-derived oversizing index (DIDO).

## Data Availability

The datasets used and analyzed during the current study are available from the corresponding author on reasonable request.

## Abbreviations

2D: Two-dimensional
3D: Three-dimensional
AF: Atrial fibrillation
AR: Aortic regurgitation
AS: Aortic stenosis
ASE: American society of echocardiography
AUC: Area under curve
AV: Aortic valve
AVA: Aortic valve area
AVAi: Aortic valve area indexed
AVC: Aortic valve calcium
BE: Balloon expandable
BMI: Body mass index
BSA: Body surface area
Ca+2: Calcification
CABG: Coronary artery bypass graft
CAD: Coronary artery disease
CHB: Complete heart block
CKD: Chronic kidney disease
CLD: Chronic lung disease
CMRI: Cardiac magnetic resonance imaging
CoVs: Cut off Values
CrCl: Creatinine clearance
CT: Computerized tomography
CV: Core valve
CVS: Cerebrovascular stroke
CW: Continuous wave
DEMOs: Demonstration models
DI: Depth of implantation
DIDO: Depth of implantation derived oversizing index
DIi: Depth of implantation indexed
DIMS: Depth of implantation to membranous septum ratio
DLZ: Device landing zone
DM: Diabetes mellitus
ECG: Electrocardiogram
EDV: End-diastolic volume
EF: Ejection fraction
ESC: European society of cardiology
EOAs: Effective orifice areas
HTN: Hypertension
ICM: Ischemic cardiomyopathy
IHD: Ischemic heart disease
IQR: Inter quartile range
IVCD: Inter ventricular conduction delay
LBBB: Left Bundle branch block
LV: Left ventricle
LVOT: Left ventricle out flow tract
LVEDD: Left ventricle end diastolic dimension
LVEDDi: Left ventricle end diastolic dimension indexed
LVESD: Left ventricle end systolic dimension
LVESDi: Left ventricle end systolic dimension indexed
MDCT: Multi detector computerized tomography
MPG: Mean pressure gradient
MS: Membranous septum
MSi: Membranous septum indexed
M-mode: Motion mode
MSID: Membranous septum to implantation depth
NB: Note Book
NCC: Non coronary cusp
NOCD: New onset conduction disturbance
NPV: Negative predictive value
PPG: Peak pressure gradient
OI: Oversizing index
PPM: Permanent pace maker
PPV: Positive predictive value
PVL: Para-valvular leakage
Pw: Pulsed wave
PWT: Posterior wall thickness,
PWTi: Posterior wall thickness indexed
QRS: QRS complex
RBBB: Right bundle branch block
RCA: Right coronary artery height
RCA i: Right coronary artery height indexed
RHD: Rheumatic heart disease
ROC: Receiver operating characteristic curve
RVSP: Right ventricular systolic pressure
SE: Self expandable
STS: Society of thoracic surgery predicted risk of mortality
SWT: Septal wall thickness
SWTi: Septal wall thickness indexed
TAVI: Transcatheter aortic valve implantation
TTE: Trans-thoracic echocardiography
VC: Vena Contracta
Vs: Versus
